# Physiological and Phytochemical Responses of *Calendula officinalis* L. to End-of-Day Red/Far-Red and Green Light

**DOI:** 10.3390/biology14080935

**Published:** 2025-07-24

**Authors:** Luisa F. Lozano-Castellanos, Giuseppina Pennisi, Luis Manuel Navas-Gracia, Francesco Orsini, Eva Sánchez-Hernández, Pablo Martín-Ramos, Adriana Correa-Guimaraes

**Affiliations:** 1 TADRUS Research Group, Department of Agricultural and Forestry Engineering, ETSIIAA, University of Valladolid, Avda. Madrid 44, 34004 Palencia, Spainpmr@uva.es (P.M.-R.); adriana.correa@uva.es (A.C.-G.); 2 Research Group on Biodiversity and Dynamics of Tropical Ecosystems-GIBDET, Faculty of Engineering Forestry, University of Tolima, Ibagué 730006, Colombia; 3 Vegetable Crops and Urban Horticulture Research Group, Department of Agricultural and Food Sciences, DISTAL, Alma Mater Studiorum—Bologna University, 40127 Bologna, Italy; giuseppina.pennisi@unibo.it (G.P.); f.orsini@unibo.it (F.O.)

**Keywords:** Calendula, supplemental light, secondary metabolism, phytochemical composition, infrared spectroscopy, GC-MS, plant physiology, marigold

## Abstract

*Calendula officinalis* L. is a widely used medicinal plant whose secondary metabolism and morphology are influenced by light. This study evaluated the effects of 2 and 4 h end-of-day (EOD) red/far-red (R:FR) and green (G) light on the growth, physiology, and phytochemical profile of hydroponically grown *C. officinalis* under a constant red/blue light background, compared with a red/blue control without EOD treatment. Morphological, physiological (gas exchange, chlorophyll fluorescence), biochemical (chlorophyll, anthocyanin), and chemical composition (attenuated total reflectance-Fourier transform infrared spectroscopy (ATR-FTIR) and Gas Chromatography-Mass Spectrometry (GC-MS)) were evaluated. EOD G 2 h enhanced photosynthetic pigments, anthocyanins, and biomass, while control plants showed higher phenolic content. EOD R:FR induced stem elongation but reduced pigment and metabolite accumulation. GC-MS revealed organ-specific metabolic specialization, with flowers displaying greater chemical diversity than leaves. EOD G favored sesquiterpene diversity in flowers, while EOD R:FR increased nitrogen-containing compounds and unsaturated fatty acids. Vibrational data supported these shifts, with spectral signatures of esters, phenolics, and lipid-related structures. Bioactive compounds, including *α*-cadinol and carboxylic acids, were identified across treatments. These findings demonstrate that EOD light modulates physiological and metabolic traits in *C. officinalis*, highlighting EOD G as an enhancer of biomass and phytochemical richness for pharmaceutical applications under controlled conditions.

## Simple Summary

Calendula, commonly known as marigold, is a valuable medicinal plant widely used in pharmaceuticals and cosmetics for its healing properties. Growing marigold under artificial lighting in controlled environments allows for year-round production, but understanding how different light colors affect plant development and beneficial compound production is essential. This study examined how short exposures—two or four hours—of green light or a combination of red and far-red light at the end of the day influence the growth, flowering, and chemical composition of marigold plants cultivated in water-based systems called hydroponics. Plants exposed to two hours of green light produced more biomass and flowers, retained higher levels of antioxidants, and accumulated a wider range of therapeutic compounds, particularly in the flowers. In contrast, red and far-red light led to taller plants but reduced both total biomass and health-promoting compounds. These findings demonstrate that adjusting light color at the end of the day can shape plant growth and compound quality, helping optimize indoor farming for the sustainable production of high-value natural substances with pharmaceutical potential.

## 1. Introduction

Artificial lighting is essential in controlled-environment agriculture, allowing precise manipulation of spectral composition, intensity, and photoperiod to optimize plant growth, productivity, and quality [1]. Advances in LED technology enable species-specific light regimes, particularly in vertical farming where natural light is limited [2]. Spectral modulation regulates processes such as photosynthesis, photomorphogenesis, flowering, and secondary metabolism, enhancing biomass production and phytochemical accumulation [3]. Multiple wavelengths often act synergistically, resulting in photosynthetic rates that surpass the additive contributions of individual spectra. These effects are maximized under controlled environments with optimized light regimes [2,4].

Plants perceive specific light wavelengths via photoreceptors that trigger gene expression and metabolism [5], coordinating key processes such as photosynthesis, morphogenesis, photoperiodic flowering, and stress adaptation [6].

Phytochromes (PhyA–PhyE) detect red (R) and far-red (FR) wavelengths (600 and 750 nm) [7]. This spectral sensitivity enables plants to detect light quality, including photoperiod and canopy shading, thereby regulating key developmental processes such as seed germination, de-etiolation, circadian entrainment, and photoperiodic flowering [8].

Red light enhances photosynthetic efficiency, due to its effective absorption by chlorophyll and high quantum yield [9]. It also promotes floral induction by activating phytochrome-mediated signaling [7], and penetrates the canopy efficiently [10]. Far-red light modulates shade avoidance responses, simulating canopy shading and promoting stem elongation, leaf expansion, and reduced branching [5].

Blue (B) and ultraviolet-A (UV-A) light (320–500 nm) are detected by phototropins, cryptochromes, and LOV/F-box/Kelch domain proteins [7]. Cryptochromes are central to light-regulated development. Blue light supports photosynthesis [10], suppresses stem elongation, enhances chlorophyll biosynthesis, and chloroplast development [11], and promotes stomatal opening, improving gas exchange and water-use efficiency [12].

Although green (G) light (500–570 nm) was long considered photosynthetically inefficient, recent evidence shows it contributes to shade avoidance, influencing hypocotyl elongation and root architecture [13,14]. Its low absorptance allows deep penetration, sustaining photosynthesis in inner mesophyll layers [9,15], and reducing stomatal conductance without affecting carbon assimilation or water-use efficiency [15].

Specific light spectra at the end of the day—termed end-of-day (EOD) lighting—modulate photoperiodic responses without altering the entire photoperiod. Typically involving FR light, it influences photoreceptor-mediated signaling during the night transition [1], and has been used to modify overall plant architecture in several species [16]. In contrast, EOD G light has been less studied; however, emerging evidence indicates that it distinctly modulates stem elongation, delays flowering in short-day species, and alters shoot-to-root allocation [17]. Species-specific responses to EOD treatments are highly variable, and systematic studies using defined R:FR ratios and green light are still limited.

Herbaceous species with pharmacological value are gaining increasing attention in artificial lighting research, beyond the traditionally studied leafy vegetables and fast-growing crops, which have dominated due to their short cycles and high responsiveness to spectral manipulation [18,19].

The genus *Calendula* (Asteraceae) includes about 25 annual and perennial herbaceous species native to the Mediterranean [20]. Among these, *C. officinalis* L. is the most studied and cultivated, valued for its pharmacological, cosmetic, and ornamental relevance. It is a facultative long-day plant (LDP) with flowering promoted—but not strictly dependent on—long photoperiods [21]. Highly adaptable to diverse climates, especially warm and humid conditions [21,22], it has been successfully cultivated in controlled-environment systems, including artificial lighting and hydroponic setups [23]. These conditions reduce contamination risks and enhance growth rate, yield, and quality in short cycles [24].

Both vegetative and reproductive organs of *C. officinalis* are valued for their bioactive compounds. Inflorescences are the principal source of secondary metabolites such as carotenoids, terpenoids, flavonoids, coumarins, and volatile compounds [20,22,25,26,27] while leaves contribute flavonols, phenolic acids, essential oils, sterols, and lipids [28,29,30], all with antibacterial, anti-inflammatory, antioxidant, antiviral, and antitumor activities [20].

Given the increasing demand for standardized phytochemical production, elucidating the influence of spectral quality on both foliar and floral tissues in *C. officinalis* remains essential for optimizing controlled cultivation practices, especially considering the commercial relevance of this species for bioactive compound extraction.

Although the application of lighting technologies in *C. officinalis* is relatively recent, studies show that R and B combinations enhance growth, biomass accumulation, and induce early flowering [3,31,32,33]. In other LDP species, although B has not been widely used in floricultural production [34], moderate intensities may attenuate the elongation effects without significantly influencing flowering induction [33,35,36]. In contrast, flowering promotion in LDPs is often associated with R:FR light combinations under low to moderate irradiance levels [37], whereas high-intensity light can exert an inhibitory effect [38].

Green light, although less studied than R, FR, or B light and only weakly absorbed by cryptochrome, has demonstrated potential to stimulate flowering in LDPs, particularly when applied at sufficiently high photon flux densities. Its involvement in shade avoidance responses makes it a suitable candidate for modulating photoperiodic signaling, particularly when applied as an EOD treatment [17,39]. However, in contrast to EOD applications of FR or R:FR light—which have been widely studied and shown to promote flowering in LDP species such as petunia, campanula, pansy, and marigold [40,41]—EOD applications of G light remain largely unexplored.

Despite this progress, no studies have evaluated the comparative effects of EOD G and R:FR light on the photomorphogenic responses of *C. officinalis*, particularly under hydroponic conditions. Previous research has focused on continuous light regimes, with little attention to short-duration EOD interventions. This study addresses this gap by exploring, for the first time, how brief EOD cues of G and R:FR light affect the growth cycle and phytochemical production of *C. officinalis* in a controlled environment. Our findings provide new insights into the species-specific responses to EOD spectral treatments and expand the applicability of precision lighting strategies in high-value medicinal plants.

We hypothesized that brief EOD spectral cues—supplied as G or R:FR light delivered after a red/blue baseline—would differentially modulate photoreceptor signaling in *C. officinalis*, leading to distinct trajectories of vegetative growth and flowering. To test this hypothesis, this study aims to examine how 2 h and 4 h EOD supplements of G or R:FR light, applied hydroponically, affect growth dynamics, developmental timing, and phytochemical production across the plant’s life cycle.

## 2. Materials and Methods

### 2.1. Growth Conditions

*Calendula officinalis* seeds, provided by the Aragon Agri-Food Research and Technology Center (CITA), Department of Plant Science (Zaragoza, Spain), were germinated in plastic trays filled with a peat and perlite mixture (70:30, *v*/*v*). Germination and growth were carried out in the vertical farming facility AlmaVFarm, managed by the Department of Agricultural and Food Sciences at the University of Bologna (Bologna, Italy). The controlled environmental conditions during the germination phase included a R:B light ratio of 70:30 (Flygrow Interlight fixtures, Flytech LED Technology, Belluno, Italy), with a photosynthetic photon flux density (PPFD) of 200 μmol m^−2^ s^−1^ under a 16 h photoperiod. Throughout the experimental cycle, the temperature averaged 24.7 °C, with a CO_2_ concentration of 555 ppm and relative humidity of 72.9%.

**Figure 1:**
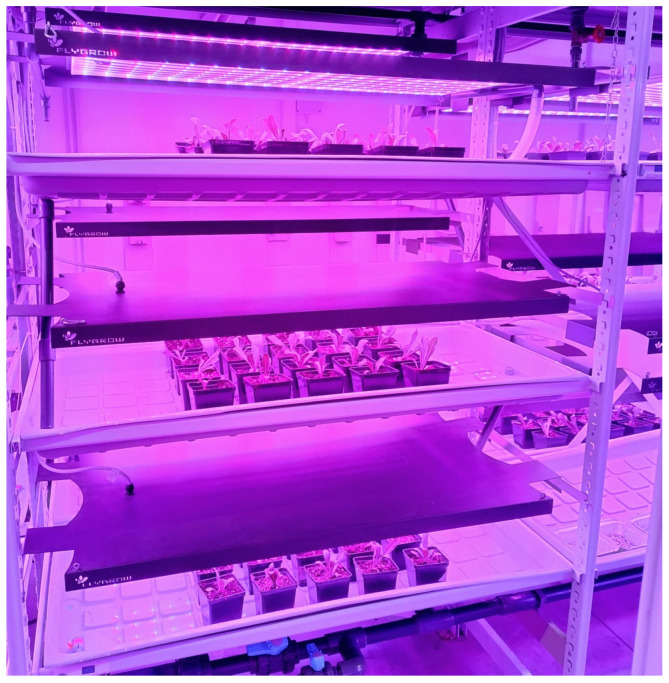
*Calendula officinalis* L. plants cultivated in the three-level ebb-and-flow hydroponic system under controlled lighting conditions (44°30′51″ N, 11°24′21″ E).

Transplanting was conducted 19 days after sowing (DAS) into individual plastic pots (9 × 9 × 10 cm) containing the same peat–perlite substrate to promote optimal root development and nutrient uptake. Plants were cultivated at a density of 70 plants m^−2^, maintaining a constant distance of 10 cm between the apical region of the canopy and the light source throughout the experimental period. The experiment was conducted as a single cultivation cycle comprising five light treatments (four EOD treatments and one control), each applied concurrently. Treatments were arranged within a three-level ebb-and-flow hydroponic system, with each level representing one independent replicate (Figure 1). Each treatment included 60 plants, divided into three replicates of 20 plants each. Within each replicate, six plants were designated as primary experimental units, while the remaining fourteen served as buffer plants.

During the first eight DAS, seedlings were manually irrigated twice daily with water. From day 9 onwards, manual irrigation continued twice daily using a modified Hoagland and Arnon nutrient solution with the following composition (g L^−1^): 11.30 KH_2_PO_4_, 1.70 NH_4_NO_3_, 1.50 NH_4_H_2_PO_4_, 33.80 K_2_SO_4_, 49.60 Mg(NO_3_)_2_, 84.10 Ca(NO_3_)_2_, 14.80 KNO_3_, 3.80 micronutrients (Mikrom), and 1.64 Fe-ethylenediaminetetraacetic acid (EDTA). The electrical conductivity of the solution was adjusted to 2.0 mS cm^−1^. Automated irrigation with the same nutrient formulation was implemented from 19 DAS, with one 10 min cycle per day for 36 days, followed by two 15 min cycles per day for 20 days, and subsequently two 20 min cycles per day for the final 24 days.

### 2.2. Light Treatments

Five LED light treatments were applied, providing a 16 h photoperiod, which included four EOD light treatments, selected for their contrasting effects on photoreceptor-mediated signaling, as previously justified in the Section 1. All treatments used a red/blue (R:B) light ratio of 70:30 and a PPFD of 252.4 ± 15.95 μmol m^−2^ s^−1^. Two treatments consisted of 12 h of R:B light followed by a 4 h EOD supplementation: one with R:FR light (30:70) at a PPFD of 53.2 ± 2.5 μmol m^−2^ s^−1^ (EOD R:FR 4 h), and the other with G light (100) at a PPFD of 51.7 ± 1.9 μmol m^−2^ s^−1^ (EOD G 4 h). Two additional treatments included 14 h of R:B light followed by a 2 h EOD light supplementation with the same spectral characteristics: R:FR light (EOD R:FR 2 h) and G light (EOD G 2 h). A control treatment received continuous R:B light for 16 h without any EOD supplementation.

PPFD was measured using a QSO quantum sensor (Apogee Instruments, Logan, UT, USA) connected to a ProCheck handheld reader (Decagon Devices Inc., Pullman, WA, USA). Measurements were taken at the plant level. PPFD values were recorded for each tray to configure the light intensity required for the treatments (Figure S1).

### 2.3. Morphological Measurements

Non-destructive measurements were conducted on the six central plants designated as primary experimental units within each replicate of every light treatment to assess vegetative and reproductive development.

Measurements were carried out at five specific time points: 27, 42, 56, 70, and 84 DAS. Vegetative development was evaluated by recording the total number of leaves and measuring stem height, as well as the length and width of fully expanded, medium-sized leaves randomly selected from each plant. Reproductive development was assessed by quantifying the number of visible flower buds and open flowers, and by measuring capitulum diameter, floret length, and floral stem length.

### 2.4. Physiological Parameters

Gas exchange and chlorophyll fluorescence were measured at 27, 42, 56, 70, and 84 DAS on the same six centrally located plants per replicate. Measurements were performed on randomly selected, fully expanded, medium-sized leaves to ensure consistency and representativeness across all treatments. A LI-COR (Lincoln, NE, USA) portable photosynthesis system (model LI-6850, version 1.4.22) equipped with a 6800-01A fluorometer (6 cm^2^ aperture) was used. The instrument’s infrared gas analyzers were zero-calibrated according to the manufacturer’s instructions, and oxygen concentration was set to 21%. The chamber was configured in broadleaf geometry (leaf width = 2.76 cm) with a 6.00 cm^2^ leaf area and a stomatal ratio (K) of 0.5. The flow rate was maintained at 600 μmol s^−1^, and the reference CO_2_ concentration was set to 850 μmol mol^−1^, while temperature and humidity controls were disabled. Actinic illumination was provided by the internal fluorometer lamp at a PPFD of 250 μmol m^−2^ s^−1^, with ~90% red and 10% blue light. Automatic CO_2_ or H_2_O matching was disabled to avoid measurement interruptions. Data were logged once gas exchange and fluorescence signals stabilized, after which the chamber was repositioned on a new leaf.

Gas exchange parameters included the net photosynthetic assimilation rate (A, µmol CO_2_ m^−2^ s^−1^), transpiration rate (E, mol H_2_O m^−2^ s^−1^), and stomatal conductance to water vapor (gsv, mol H_2_O m^−2^ s^−1^), which together characterize photosynthetic performance and water-use dynamics. In parallel, chlorophyll fluorescence parameters included the electron transport rate (ETR, µmol electrons m^−2^ s^−1^), the maximum quantum efficiency of photosystem II (PSII) in the light-adapted state (Fv′/Fm′), and the effective quantum yield of PSII (ΦPSII). These metrics provide insight into the functional status and photochemical efficiency of PSII under ambient light conditions.

### 2.5. Spectral and Fluorescence Imaging Analysis

Pigment content and related parameters were assessed using the PlantExplorer PRO+ system (PhenoVation, Wageningen, The Netherlands) through non-destructive imaging of the plant canopy at 27, 42, 56, 70, and 84 DAS on the six main plants. This system is designed to measure pulse amplitude modulated (PAM) fluorescence (including OJIP induction curves), multispectral reflectance, and color. It was operated in open protocol mode with a 3.1-megapixel high-resolution setting under dark- and light-adapted conditions, using a PAM configuration. The parameters analyzed included chlorophyll and anthocyanin content. Data were acquired and processed with the Crop Reporter Phenotyping imaging station software (PhenoVation B.V., Version 5.8.0 beta 64b).

### 2.6. Biomass

At 84 DAS, destructive sampling was conducted to determine biomass accumulation. Four primary plants per replicate were harvested, and leaves, flowers, buds, and stems were separated and weighed immediately to record fresh weight using a Kern analytical balance ABJ 80-4NM+963-101 (Kern & Sohn GmbH, Balingen, Germany). All samples were then placed in an SLW 400 drying oven (POL-EKO^®^ sp.k., Wodzisław, Poland) at 60 °C for five days and subsequently weighed to determine dry weight.

### 2.7. Chemical Composition Analysis

The two remaining plants per replicate were designated for the chemical composition analysis of leaves and flowers. Approximately 70 g of fresh tissue per organ (leaves and flowers) were collected and dried to constant weight in a forced-air oven (SLW 400; Pol-Eko, Wodzisław Śląski, Poland) at 52.5 °C for approximately four days. This process resulted in a mass reduction of 91.50 ± 1.40% for leaves and 81.86 ± 3.70% for flowers, corresponding to moisture loss. Dried tissues were then ground to a fine powder, homogenized, and passed through a 1 mm mesh sieve. The powders were stored separately in 250 mL plastic bottles.

For extraction, defined amounts of dried material from each treatment were mixed with HPLC-grade methanol (Sigma Aldrich Química S.A., Madrid, Spain) and distilled water in a 1:1 volume ratio (50 mL each). The quantities of dried tissue used per treatment were as follows: control (25.1 g flowers, 9.4 g leaves), EOD G 2 h (23.1 g flowers, 7.6 g leaves), EOD G 4 h (27.6 g flowers, 7.9 g leaves), EOD FR 2 h (31.7 g flowers, 8.3 g leaves), and EOD FR 4 h (26.0 g flowers, 10.78 g leaves). Each mixture was heated at 50 °C for 30 min and then sonicated using a probe-type ultrasonicator (UIP1000 hdT; Hielscher Ultrasonics, Teltow, Germany) in alternating cycles of 10–15 min of sonication followed by 5–10 min of rest, to maintain the temperature between 30 and 60 °C. The resulting extracts were decanted, filtered through Whatman No. 1 paper, and further passed through a 0.22 µm membrane filter. They were then centrifuged at 9000 rpm for 15 min. The supernatants were rapidly frozen at −80 °C in a TwinCool ULT freezer (Haier Biomedical, Qingdao, China) and subsequently freeze-dried in a Teslar lyoQuest HT-40 freeze-drier (Beijer Electronics Products AB, Malmö, Sweden).

For attenuated-total-reflectance Fourier-transform infrared (ATR-FTIR) analysis, the freeze-dried extracts were analyzed using a Nicolet iS50 FTIR spectrometer (Thermo Scientific, Waltham, MA, USA) equipped with a diamond ATR module. Spectra were acquired by co-adding 64 scans over the spectral range of 400–4000 cm^−1^ at a resolution of 1 cm^−1^.

For gas chromatography–mass spectrometry (GC–MS) analysis, the freeze-dried extracts of flowers and leaves from each treatment were reconstituted in HPLC-grade methanol to a final concentration of 5 mg·mL^−1^ and subsequently filtered. The analysis was carried out at the Research Support Services (SSTTI) of the University of Alicante (Alicante, Spain), using a system from Agilent Technologies (Santa Clara, CA, USA) comprising a model 7890A gas chromatograph coupled to a model 5975C quadrupole mass spectrometer. Chromatographic conditions were as follows: injection volume of 1 µL; injector temperature set at 280 °C in splitless mode; initial oven temperature of 60 °C held for 2 min, followed by a ramp of 10 °C·min^−1^ to a final temperature of 300 °C, which was maintained for 15 min. Compound separation was achieved using an HP-5MS UI column (30 m × 0.250 mm i.d., 0.25 µm film thickness; Agilent Technologies). The mass spectrometer operated with an electron impact ionization source at 70 eV, with source and quadrupole temperatures of 230 and 150 °C, respectively.

Compound identification was based on the comparison of mass spectra and retention times with those of authentic standards and by computer matching against the National Institute of Standards and Technology (NIST11) library. Instrument calibration was performed using Test Mixture 2 for apolar capillary columns (Grob, Supelco 86501, Mindelheim, Germany) and perfluorotributylamine (PFTBA) tuning standards to ensure analytical accuracy.

### 2.8. Statistical Analysis

Statistical analyses were performed using Python version 3.11, an open-source programming language. Morphological and reproductive data of *C. officinalis* were tested for normality using the Shapiro–Wilk test, and for homogeneity of variances using Levene’s test. Both assumptions were met. Subsequently, a two-way analysis of variance (ANOVA) was conducted to determine the effects of the light treatments, time (DAS), and their interaction. Statistical significance was determined at *p* ≤ 0.05. When significant effects were found for the factor “treatment”, pairwise comparisons between treatment means were conducted using Tukey’s honest significant difference (HSD) test. Graphs displaying the mean ± standard error of the mean (SEM) were also generated using Python.

## 3. Results

### 3.1. Morphological and Floral Development

The two-way ANOVA revealed that all four morphological variables—leaf number, stem length, leaf width, and leaf length—were significantly influenced by light treatment, time (DAS), and their interaction (*p* ≤ 0.05 for all; Table 1).

**Figure 2:**
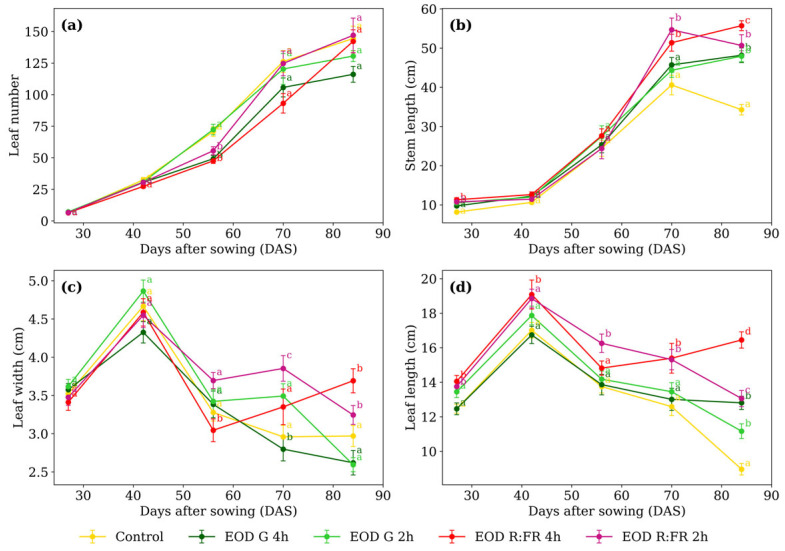
(**a**) Leaf number, (**b**) stem length, (**c**) leaf width, and (**d**) leaf length of *C. officinalis* under different light spectra across five measurement periods: 27, 42, 56, 70, and 84 days after sowing (DAS). Data represent means ± standard error of the mean (SEM). Treatments sharing the same letter within each DAS are not significantly different according to Tukey’s HSD test (α = 0.05).

Significant differences in leaf number (Figure 2a) among treatments were detected only at 56 DAS. EOD G 4 h, EOD R:FR 2 h, and EOD R:FR 4 h had a 29.1% reduction in leaf production compared to the remaining treatments. Regarding stem length (Figure 2b), at 27 DAS, plants under EOD G 2 h, EOD R:FR 2 h, and EOD R:FR 4 h represented a 27.7% elongation increase compared to the control and EOD G 4 h. At 70 DAS, EOD R:FR 2 h and EOD R:FR 4 h continued to promote elongation, with an average increase of 31.2%. By 84 DAS, EOD R:FR 4 h induced the greatest stem length, followed by EOD R:FR 2 h, EOD G 4 h, and EOD G 2 h, all exceeding the control by 47.6% on average.

At 56 DAS, EOD R:FR 4 h resulted in the narrowest leaves, representing an 11.6% reduction compared to the average of the other treatments (Figure 2c). By 70 DAS, EOD R:FR 2 h showed the widest leaves, while EOD G 4 h recorded the narrowest, with a difference of 37.5%. At 84 DAS, EOD R:FR 2 h and EOD R:FR 4 h showed the greatest values, averaging 3.47 cm, which was 27.1% higher than the remaining treatments.

Leaf length (Figure 2d) showed at 27 DAS, R:FR treatments exceeding the other treatments by 9.4%. At 42 DAS, only EOD R:FR 4 h differed, with a 9.1% increase. At 56 DAS, EOD R:FR 2 h reached 16.4% longer than the group mean. At 70 DAS, R:FR treatments surpass the rest by 18.5%. At 84 DAS, EOD R:FR 4 h produced the longest leaves, 83.3% longer than the control.

Concerning the flowering phase, flower bud number, flower number, floret length, capitulum diameter, and floral stem length exhibited significant main effects of treatments and time (*p* < 0.0001 in all cases), with additional interaction effects (*p* < 0.05), demonstrating that the response to light spectra varied across developmental stages (Table 1).

**Figure 3:**
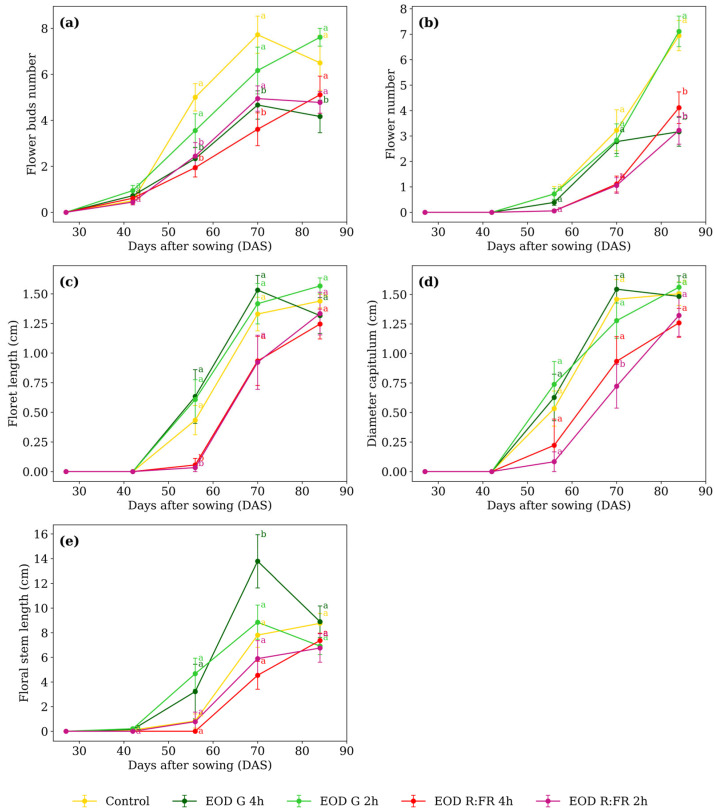
(**a**) Flower bud number, (**b**) flower number, (**c**) floret length, (**d**) capitulum diameter, and (**e**) floral stem length of *C. officinalis* under different light spectra across five measurement periods: 27, 42, 56, 70, and 84 DAS. Data represent means ± SEM. Treatments sharing the same letter within each DAS are not significantly different according to Tukey’s HSD test (α = 0.05).

At 56 DAS, the number of flower buds (Figure 3a) averaged 4.3 in the control and EOD G 2 h. At 70 DAS, EOD G 4 h and EOD R:FR 4 h showed 4.1 buds on average. By 84 DAS, EOD G 4 h remained lower while the other treatments ranged from 4.7 to 7.6 buds.

Flower number (Figure 3b) at 70 DAS, control, EOD G 2 h, and EOD G 4 h averaged 2.9 flowers. At 84 DAS, control and EOD G 2 h averaged 7.0 flowers, whereas EOD G 4 h, EOD R:FR 2 h, and EOD R:FR 4 h averaged 3.5, indicating a 100% increase in flower production. Floret length (Figure 3c) differed only at 56 DAS. EOD R:FR 2 h and EOD R:FR 4 h averaged 0.045 cm, while control, EOD G 2 h, and EOD G 4 h averaged 0.558 cm. This corresponds to a 91.9% reduction in floret elongation under R:FR light.

At 56 DAS, capitulum diameter (Figure 3d) ranged from 0.08 to 0.74 cm. At 70 DAS, EOD R:FR 2 h showed the lowest value, with 0.72 cm, while the remaining treatments averaged 1.30 cm. This reflects a 44.7% reduction under EOD R:FR 2 h. At 84 DAS, values ranged from 1.26 to 1.56 cm, with no significant differences among treatments. Floral stem length (Figure 3e) differed only at 70 DAS. EOD G 4 h showed 13.7 cm, while the remaining treatments averaged 6.8 cm, representing a 103.6% increase under EOD G 4 h. At 84 DAS, values ranged from 6.7 to 8.9 cm, with no significant differences among treatments.

### 3.2. Physiological Parameters

All gas exchange and chlorophyll fluorescence parameters showed statistically significant interactions between light treatment and developmental stage, with the exception of stomatal conductance (gsw), for which only the main effects of treatment and time were significant (Table 2).

**Figure 4:**
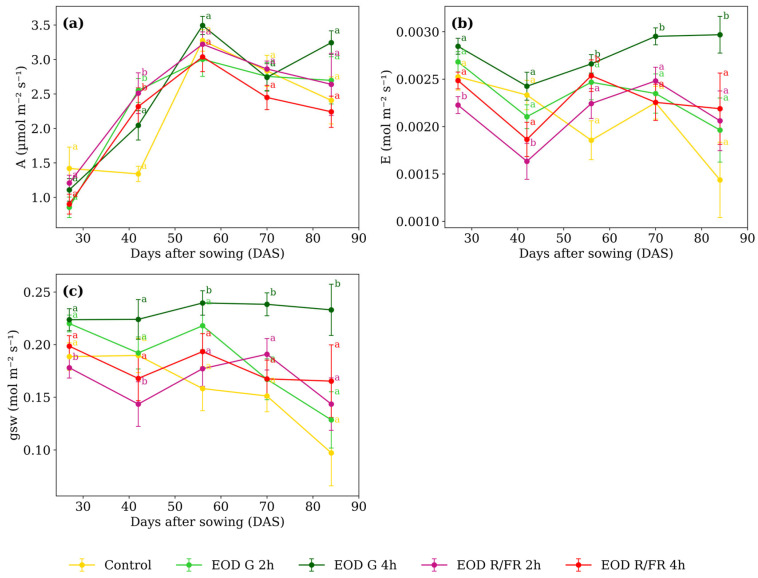
(**a**) Net photosynthetic assimilation rate, (**b**) transpiration rate, and (**c**) stomatal conductance to water vapor of *C. officinalis* under different light spectra across five measurement periods: 27, 42, 56, 70, and 84 DAS. Data represent means ± SEM. Treatments sharing the same letter within each DAS are not significantly different according to Tukey’s HSD test (α = 0.05).

Differences in A (Figure 4a) among treatments were minimal at most developmental stages. However, at 42 DAS, EOD G 4 h and control exhibited lower values, averaging 1.69 µmol m^−2^ s^−1^, while the other treatments averaged 2.46 µmol m^−2^ s^−1^, corresponding to a 31.2% increase under short-duration G and R:FR light.

At 27 DAS, E (Figure 4b) was lowest under EOD R:FR 2 h, showing a 15.6% reduction compared to the average of the other treatments. A similar trend was observed at 42 DAS, where EOD R:FR 2 h showed a 25.1% reduction relative to the group average. At 56 DAS, EOD G 4 h and EOD R:FR 4 h showed elevated transpiration compared to the other treatments. At 70 and 84 DAS, EOD G 4 h consistently presented the highest transpiration values among all treatments.

From 56 DAS onwards, a marked increase in gsw (Figure 4c) was observed under EOD G 4 h (reaching 0.239, 0.238, and 0.233 mol m^−2^ s^−1^ at 56, 70, and 84 DAS, respectively) At 56 DAS, the average of the other treatments was 0.187, representing a 28.3% increase under EOD G 4 h. This trend persisted at 70 and 84 DAS, where EOD G 4 h exceeded the group averages (0.169 and 0.133 mol m^−2^ s^−1^, respectively) by 40.6% and 75.5%. In contrast, at earlier stages, EOD R:FR 2 h showed the lowest values, with 0.178 at 27 DAS and 0.143 at 42 DAS, compared to averages of 0.207 and 0.193 recorded for the other treatments.

**Figure 5:**
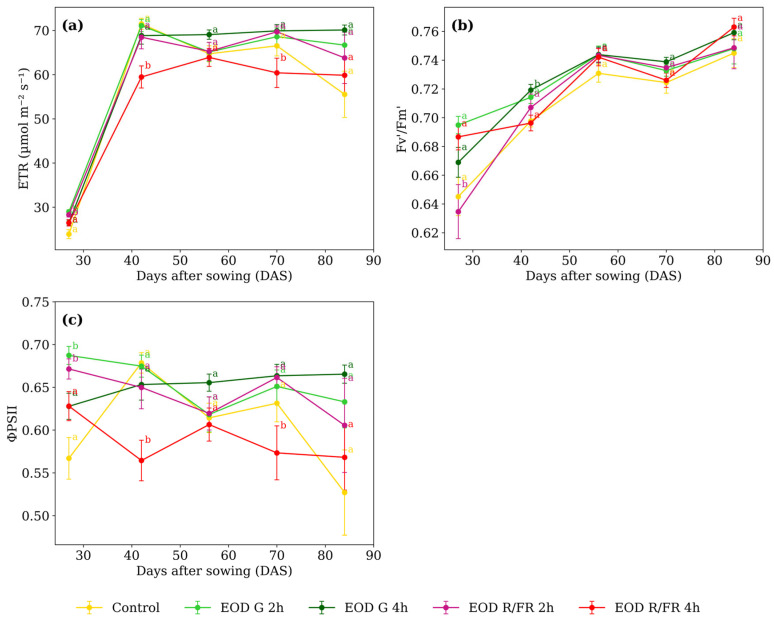
(**a**) Electron transport rate, (**b**) maximum quantum efficiency of PSII, and (**c**) effective quantum yield of PSII of *C. officinalis* under different light spectra across five measurement periods: 27, 42, 56, 70, and 84 DAS. Data represent means ± SEM. Treatments sharing the same letter within each DAS are not significantly different according to Tukey’s HSD test (α = 0.05).

At 27 DAS, EOD G 2 h and EOD R:FR 2 h exhibited the highest ETR (Figure 5a), with an average of 28.63 µmol m^−2^ s^−1^. At 42 DAS, EOD R:FR 4 h showed the lowest value, 15.0% below the average of the other treatments. A similar pattern was observed at 70 DAS, where EOD R:FR 4 h again presented the lowest ETR, 11.4% lower than the average of the remaining treatments.

At 27 DAS, EOD R:FR 2 h exhibited the lowest Fv’/Fm’ value (0.635), representing a 5.8% reduction compared to the average of the other treatments (Figure 5b). At 42 DAS, EOD G 4 h showed the highest value (0.719), slightly above the control (0.698). From 56 DAS onwards, no substantial differences were observed, with values ranging narrowly from 0.743 to 0.763 across all treatments.

At 27 DAS, ΦPSII values were higher in EOD G 2 h and EOD R:FR 2 h, averaging 0.679 (Figure 5c). At 42 DAS, EOD R:FR 4 h showed the lowest value (0.564), which was 16.2% lower than the average of the other treatments. A similar reduction was observed at 70 DAS, where it again presented the lowest value (0.573). From 56 DAS, values ranged from 0.606 to 0.665, and no major differences were observed among treatments. At 84 DAS, ΦPSII values were also similar, ranging from 0.527 in the control to 0.665 in EOD G 4 h.

### 3.3. Spectral and Fluorescence Imaging Analysis

**Figure 6:**
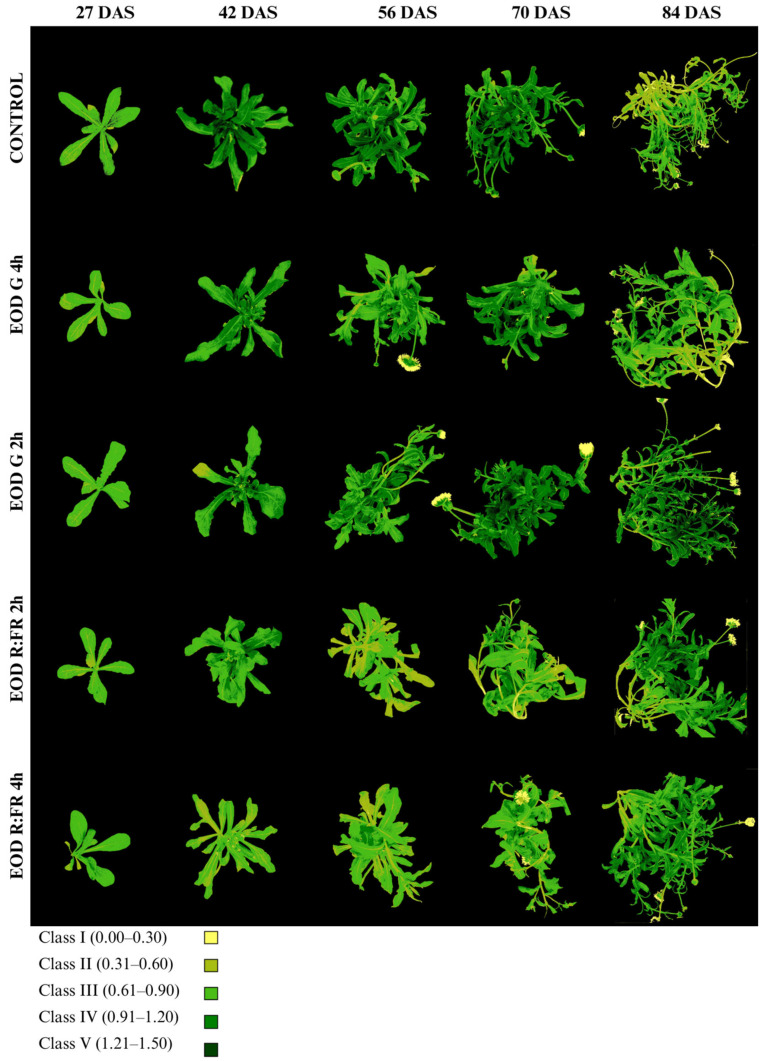
Evolution of chlorophyll accumulation in *C. officinalis* at 27, 42, 56, 70, and 84 DAS under different end-of-day light treatments.

The chlorophyll index (Chl-Idx) and class distribution are presented in Figure 6, Table 3 and Table S1. The Chl-Idx values were categorized into five classes based on ranges: class I (0.00–0.30), class II (0.31–0.60), class III (0.61–0.90), class IV (0.91–1.20), and class V (1.21–1.50).

At 27 DAS, all treatments exhibited a predominance of class III, with the proportion of plant area ranging from 76.7% in EOD R:FR 2 h to 84.6% in EOD G 2 h. The highest Chl-Idx at this stage was recorded in EOD G 2 h (0.81 ± 0.12), followed by EOD R:FR 4 h (0.79 ± 0.14), while the control showed a lower index of 0.70 ± 0.11. From 42 DAS onward, a progressive shift in class distribution was observed, with increasing proportions of classes III, IV, and V, particularly under the control, EOD G 2 h, and EOD G 4 h treatments. At 42 DAS, the control plants presented the highest Chl-Idx (1.09 ± 0.25), with 49.1% of the area corresponding to class IV and 30.7% to class III. In contrast, EOD R:FR 4 h exhibited the lowest index (0.64 ± 0.15), with most of the leaf area distributed between class II (55.4%) and class III (39.9%).

At 56 DAS, the Chl-Idx remained elevated under the control (1.12 ± 0.26) and EOD G 2 h (1.01 ± 0.26) treatments. In contrast, EOD R:FR 2 h and EOD R:FR 4 h maintained lower Chl-Idx values (0.69 ± 0.15 and 0.76 ± 0.16, respectively), although they retained a high proportion of class III (52.8% and 65.3%, respectively). At 70 DAS, the highest Chl-Idx was observed in EOD G 2 h (1.16 ± 0.34), with 51.7% of the leaf area in class IV and 21.6% in class V. The control and EOD G 4 h treatments also displayed high indices (1.11 ± 0.26 and 1.09 ± 0.28, respectively), whereas EOD R:FR 2 h and EOD R:FR 4 h remained comparatively lower (0.82 ± 0.26 and 0.81 ± 0.23, respectively).

By 84 DAS, EOD G 2 h retained the highest Chl-Idx (1.08 ± 0.34), with considerable portions of the leaf area in class III (25%), class IV (41.5%), and class V (17.5%). The control group showed a decrease in Chl-Idx to 0.73 ± 0.24, with most of the area falling within class II (36.2%) and class III (44.7%). EOD R:FR 2 h and EOD R:FR 4 h presented moderate values (0.97 ± 0.30 and 0.96 ± 0.25, respectively), while EOD G 4 h displayed an intermediate value (0.81 ± 0.27), with a predominance of class III (50.3%). Overall, EOD G 2 h consistently promoted higher chlorophyll accumulation throughout the growth period, whereas the control, EOD R:FR 4 h, and EOD R:FR 2 h were associated with lower Chl-Idx values and reduced proportions of high-index classes.

**Figure 7:**
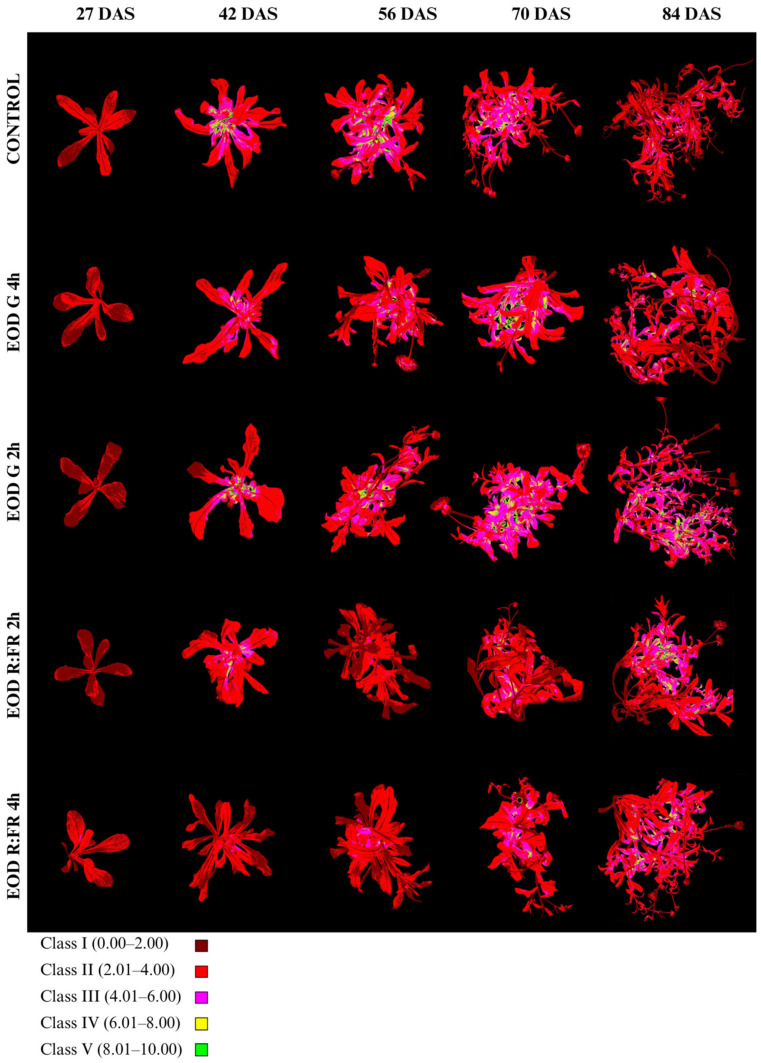
Evolution of anthocyanin accumulation in *C. officinalis* at 27, 42, 56, 70, and 84 DAS under different end-of-day light treatments.

Anthocyanin index (Ari_Idx) and class distribution are summarized in Figure 7, Table 4 and Table S2. Ari_Idx values were classified into five categories: class I (0.00–2.00), class II (2.01–4.00), class III (4.01–6.00), class IV (6.01–8.00), and class V (8.01–10.00).

At 27 DAS, all treatments showed a clear predominance of class I, ranging from 56.6% in EOD G 2 h to 80.8% in EOD R:FR 2 h. The lowest Ari_Idx was recorded in EOD R:FR 2 h (1.70 ± 0.50), followed by EOD G 4 h (1.85 ± 0.50) and the control (1.90 ± 0.52), whereas EOD G 2 h and EOD R:FR 4 h reached higher values of 1.99 ± 0.57 and 1.91 ± 0.67, respectively. By 42 DAS, there was a marked increase in all treatments, particularly in the control (3.62 ± 2.61), which presented the highest index and a dominant proportion of class II (59.0%) and class III (23.1%). EOD G 2 h and EOD G 4 h showed similarly elevated indices of 3.44 ± 1.67 and 3.01 ± 1.25, respectively, with more balanced distributions among classes II–IV. In contrast, EOD R:FR 2 h and EOD R:FR 4 h exhibited lower values of 2.66 ± 1.02 and 1.89 ± 0.66, respectively, and a higher proportion of class I (21.7% and 62.8%).

At 56 DAS, the highest Ari_Idx remained in the control (3.66 ± 1.55), with classes II and III comprising the majority of the leaf area (56.9% and 24.1%, respectively). EOD G 2 h and EOD G 4 h continued to show elevated indices of 3.19 ± 1.35 and 2.94 ± 1.26, while EOD R:FR 2 h and EOD R:FR 4 h had 1.97 ± 0.66 and 2.22 ± 0.80.

At 70 DAS, the highest anthocyanin content was observed in EOD G 2 h (3.92 ± 1.60), followed by EOD G 4 h (3.67 ± 1.60) and the control (3.34 ± 1.37), each with over 45% of the area in class II and notable increases in class III. In contrast, EOD R:FR 2 h and EOD R:FR 4 h showed lower indices of 2.47 ± 1.23 and 2.93 ± 1.30, respectively, with a large portion of leaf area in classes I and II.

By 84 DAS, EOD G 2 h maintained the highest Ari_Idx (3.71 ± 1.73), with 31.0% and 43.2% of the area in classes III and II, respectively. The control treatment showed a reduced index of 2.48 ± 1.15, with over half of the area in class II (52.2%). EOD R:FR 2 h and EOD R:FR 4 h displayed moderate indices of 3.26 ± 1.61 and 3.21 ± 1.37, respectively. EOD G 4 h presented an intermediate value of 2.69 ± 1.37. Overall, anthocyanin accumulation was enhanced under EOD G 2 h across most of the stages, while EOD R:FR treatments consistently induced the lowest pigment indices, particularly at earlier stages.

### 3.4. Biomass

Fresh biomass distribution (Table 5) revealed that total fresh weight was highest under EOD R:FR 2 h (629.64 g), followed by EOD G 2 h (600.86 g) and EOD R:FR 4 h (565.81 g). Among the individual components, only stem fresh weight differed significantly among treatments (*p* = 0.009), with EOD R:FR 2 h promoting greater stem development. In this treatment, stems accounted for 57.6% of the total fresh biomass, leaves for 39.0%, and reproductive structures (buds and flowers) for less than 4%. Under EOD G 2 h, biomass allocation was more balanced, with 47.6% assigned to leaves, 47.8% to stems, and 4.6% to reproductive organs. Although no significant differences were detected in leaf, bud, or flower fresh weights (*p* > 0.05), EOD G 2 h consistently showed the highest mean values for leaves, flowers, and buds.

A similar trend was observed in dry biomass distribution (Table 5). In EOD G 2 h, dry biomass was distributed as 45.2% in leaves, 46.6% in stems, and 8.2% in reproductive structures. In contrast, EOD R:FR 2 h allocated 37.7% to leaves, 55.9% to stems, and 6.4% to reproductive parts. While dry bud and flower weights did not differ significantly (*p* = 0.344 and 0.062, respectively), EOD G 2 h again presented the highest mean values.

### 3.5. Chemical Composition Analysis

The infrared spectra of hydromethanolic extracts from leaves and flowers (Table 6, Figures S2 and S3) revealed a broad O–H stretching band (~3294–3393 cm^−1^) in all leaf samples and also in flower samples from the control and EOD R:FR 2 h treatments, indicating the presence of hydroxyl-containing compounds such as polyphenols and water in both organs. The C–H asymmetric and symmetric stretching bands (~2917 and ~2849 cm^−1^) were present in most treatments, except in the control and EOD G treatments, where the ~2849 cm^−1^ band was absent. The C=O stretching band (~1730–1745 cm^−1^), associated with esters, was observed in all samples except EOD G 4 h leaves. The C=C stretching band (~1599–1633 cm^−1^), related to aromatic rings, was consistently present across treatments.

In the fingerprint region (1500–500 cm^−1^), flowers exhibited a higher number of bands. Notably, C–O vibrations associated with flavonoids and glycosides (~1049–1011 cm^−1^), as well as bands between ~1232 and 1144 cm^−1^ related to ester and glycosidic linkages, were exclusively detected in flowers. Similarly, multiple out-of-plane C–H bending bands (976–819 cm^−1^), characteristic of aromatic structures, were also confined to floral tissues. In contrast, CH_2_ and CH_3_ deformation bands (1322–1352 cm^−1^), along with ring deformation and C–H wagging bands (667–534 cm^−1^), were predominantly observed in leaves. Skeletal deformation at 423 cm^−1^ was detected only in leaf EOD G light treatments.

**Figure 8:**
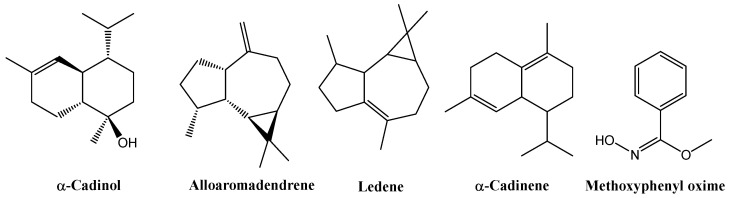
Chemical structures of the main chemical compounds identified in *C. officinalis* hydromethanolic leaf extract.

GC–MS analysis of the leaf extracts (Tables S3–S7 and Figures S4–S8) revealed a consistent terpenoid core across all treatments. All samples contained *α*-cadinol (2.91% in the control, 9.55% in EOD G 2 h, 11.00% in EOD G 4 h, 8.61% in EOD R:FR 2 h, and 23.27% in EOD R:FR 4 h), alloaromadendrene (2.63%, 4.74%, 2.49%, 2.24%, and 2.69%, respectively), ledene (5.57%, 4.52%, 5.39%, 6.17%, and 7.05%), *δ*-cadinene (1.53%, 1.33%, 1.96%, 1.54%, and 2.31%), and methoxyphenyl oxime (52.09%, 51.31%, 31.64%, 45.32%, and 37.13%) (Figure 8).

Each treatment displayed specific terpenoids. The control extract included cedrene-V6 (0.52%) and *β*-panasinsene (1.59%). EOD G 4 h showed six exclusive compounds: 2-isopropyl-5-methyl-9-methylene-bicyclo[4.4.0]dec-1-ene (3.89%), longifolene (1.35%), aromadendrene (1.08%), *α*-calacorene (0.66%), 8-epi-*γ*-eudesmol (0.48%), and megastigmatriene (0.42%). In EOD G 2 h, only *β*-guaiene (1.23%) was identified. EOD R:FR 2 h contained isoaromadendrene epoxide (1.44%) and ledol (1.39%), while EOD R:FR 4 h featured only *β*-humulene (1.39%).

Lipid composition varied notably. Methyl stearate and other fatty acid methyl esters were generally present, except in EOD G 4 h, where long-chain lipids were nearly absent.

Nitrogen-containing metabolites also differed. The control extract was enriched in aminated phenolics and aromatic oximes. EOD G 4 h contained pyrrole derivatives, benzodiazepines, and simple heterocycles, while EOD G 2 h included complex alkaloids such as carbazoles, quinolines, and indoles. EOD R:FR 2 h showed the presence of 13H-dibenzocarbazole and phenanthrolines, whereas EOD R:FR 4 h was characterized by pyridopyrimidines, piperazines, and amino acid esters.

Sulfur-containing compounds were treatment-specific: sulfonyl derivatives in the control, triazolethiones and isothiocyanates in EOD G 4 h, and thiophenes conjugated with pyrroles in EOD G 2 h.

**Figure 9:**
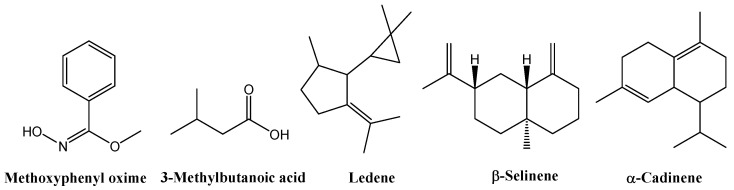
Chemical structures of the main chemical compounds identified in *C. officinalis* hydromethanolic flower extract.

GC–MS analysis of flower extracts (Tables S8–S12, Figures S9–S13) revealed a set of compounds consistently present across all treatments, including methoxyphenyl oxime (25.82% in the control, 8.53% in EOD G 2 h, 29.50% in EOD G 4 h, 8.65% in EOD R:FR 2 h, and 18.96% in EOD R:FR 4 h), 3-methylbutanoic acid (0.85%, 0.84%, 1.31%, 1.49%, and 1.30%, respectively), and ledene (4.83%, 7.77%, 5.84%, 5.53%, and 7.00%). Two related derivatives of *β*-selinene or decahydro-4a-methyl-1-methylene-7-(1-methylethenyl)-naphthalene were also detected across all samples: isomer [4aR-(4aα,7α,8aβ)] and (4aR-trans) isomer at 5.89%, 6.39%, 5.17%, 5.59%, 5.28%. Additionally, the *β*-cadinene was consistently present at 1.67%, 2.48%, 1.76%, 1.63%, and 1.78% (Figure 9).

Among sesquiterpenes, α-cadinol and alloaromadendrene were detected in the control (4.56% and 2.21%, respectively), EOD G 2 h (6.10% and 1.86%), EOD G 4 h (18.99% and not detected), EOD R:FR 2 h (not detected and 2.56%), and EOD R:FR 4 h (21.04% and 1.90%).

Nitrogen-containing compounds, including acetamide and hydroxyacetone, showed combined contents of 12.64% in the control, 14.36% in EOD R:FR 2 h, and 9.39% in EOD R:FR 4 h but were markedly reduced or absent in EOD G treatments.

Aliphatic esters and fatty acid derivatives such as methyl stearate and methyl hexadecanoate were present at 0.48% in the control, 0.88% in EOD G 4 h, and 2.53% in EOD R:FR 4 h, but were undetected in EOD G 2 h.

Within the oxygenated cyclic compound family, butyrolactone and tiglic acid contributed 0.69% in the control, 0.22% in EOD G 4 h, 1.42% in EOD R:FR 2 h, and 1.75% in EOD R:FR 4 h, but were absent in EOD G 2 h.

Decahydronaphthalene derivatives and structurally related terpenoids, such as α-selinene and 7-acetyl-2-hydroxy-2-methyl-5-isopropylbicyclo[4.3.0]nonane, were shared by most treatments and accounted for 4–6% of the total area in the control and EOD G 2 h.

## 4. Discussion

### 4.1. Morphological Responses to EOD Light Treatments

Plants exposed to EOD R:FR treatments displayed classic shade avoidance traits, most notably enhanced stem elongation compared to EOD G and control. This response became increasingly evident as plants approached reproductive development, highlighting a developmental timing component. A comparable pattern was reported in a previous study [42] in which *C. officinalis* exposed to FR-enriched conditions exhibited greater stem elongation, accompanied by a tendency toward lower chlorophyll content in the leaves, consistent with the results of this investigation. Additionally, EOD FR treatment markedly increased plant height, a response well documented in other species such as *Solanum lycopersicum* L., where brief EOD pulses induced elongated and more slender phenotypes [43]. Similar effects have been reported for *Chrysanthemum morifolium* Ramat. ex Hemsl., *Lactuca sativa* and *Helianthus annuus* L., both members of the Asteraceae family, like *C. officinalis*. EOD-FR light has been effectively employed to induce stem elongation in these species, a commercially valuable trait in ornamentals [44,45]. The similarity in response among Asteraceae members points to a conserved phytochrome-mediated mechanism regulating growth under modified light conditions in response to EOD light.

Contrary to expectations, plants exposed to EOD R:FR did not exhibit enhanced reproductive development. A low R:FR ratio is typically associated with accelerated flowering in shade-avoiding species, functioning as an adaptive mechanism to ensure reproductive success under competitive conditions [45], but these adaptations often reduce overall plant productivity. Plants exposed to EOD G and the control light exhibited more advanced reproductive development despite minimal morphological changes. A similar pattern was reported in *Arabidopsis thaliana* (L.) Heynh., where simulated shade conditions triggered early flowering with limited leaf expansion and branching [46], and in *Coriandrum sativum* L., where a moderate addition of green light to R:B LED spectra doubled fresh biomass production without triggering the shade-avoidance trait [47]. Since G wavelengths exert a weak influence on phytochrome balance and do not induce classical shade-avoidance responses [15], their role in promoting flowering may depend on alternative regulatory mechanisms, possibly activated at high photon flux densities in some LDP species [39].

### 4.2. Physiological Responses to EOD Light Treatments

Despite morphological differences, *C. officinalis*’ core physiological processes were generally stable under EOD treatments, with some nuanced variations. A progressive increase in A was observed, without statistically significant differences among the EOD light treatments. This trend is likely due to the gradual maturation of the photosynthetic apparatus rather than direct spectral effects. Comparable results were reported by Groen (1973) [48], who found that although increased light intensity during growth generally enhanced photosynthetic capacity in *C. officinalis*, treatment differences were often negligible [48]. In some cases, leaves developed under intermediate light levels exhibited comparable or higher photosynthetic rates than those exposed to full sunlight, highlighting the complexity of photosynthetic acclimation. These findings suggest that, in *C. officinalis*, factors such as leaf developmental stage and cumulative light exposure may exert a stronger influence on carbon assimilation than EOD light quality alone. Supporting this, Davodipour et al. (2025) demonstrated that the highest A values in *C. officinalis* occurred under R:B light in the absence of salinity stress [49]. Given that all plants were grown under fixed R:B light, its dominant influence likely overrode EOD light effects.

Current evidence indicates that the R:FR light ratio does not consistently lower E or gsw; in fact, several studies have reported increased water loss under FR enrichment. In *Ocimum basilicum* L., supplemental FR light elevated the stomatal aperture area, enhancing evapotranspiration and water use [50]. Similarly, in *L. sativa* L., FR-enriched conditions increased stomatal density, size, E, and gsw, particularly when the spectral ratio strongly favored FR [51]. In *C. officinalis*, plants exposed to EOD G for 4 h exhibited moderately high transpiration and stomatal conductance, while those under EOD R:FR 2 h and control treatments consistently showed the lowest values. This pattern mirrors findings in *Salvia officinalis* L., where shorter-wavelength light, such as B light or combined R:B, promoted higher transpiration rates and wider stomatal apertures, whereas higher R light reduced water loss by narrowing stomata [52]. Similarly, in *L. sativa*, green light reduced water loss and maintained photosynthetic activity, suggesting a regulatory effect on stomatal aperture [53].

Chlorophyll fluorescence parameters showed limited variation among EOD light treatments. In *C. officinalis*, previous observations under natural sunlight indicated that ETR is primarily influenced by light intensity, with higher values in full sunlight than shade-grown plants [54]. This supports the notion that *C. officinalis* may be more responsive to overall irradiance than to changes in light quality alone. In our study, EOD R:FR 4 h led to a slight reduction in ETR, possibly due to an imbalance favoring photosystem I (PSI) excitation, which can increase cyclic electron flow while suppressing linear E [55,56]. This mechanism has been described in other species, such as *L. sativa*, where moderate FR addition improves E and growth, but excessive FR—especially under high background light—can reduce photosynthesis [56]. Conversely, G light, although less efficiently absorbed at low PPFD, penetrates deeper into leaf tissues at high PPFD, supporting inner chloroplasts’ excitation [9]. This may explain why ETR values under G light treatments in *C. officinalis* remained comparable to those under EOD R:FR, despite differences in spectral composition.

Similar to E, *C. officinalis* has shown that high levels of FR light tend to reduce Fv/Fm (maximum quantum efficiency in dark-adapted conditions), indicating potential stress or downregulation of PSII [33]. A similar effect in *L. sativa* can be inferred for Fv’/Fm’ under actinic light, where excessive FR may impair PSII efficiency due to an excitation imbalance between PSI and PSII [57]. In contrast, moderate FR can improve light distribution between the two photosystems, enhancing overall photochemical performance. ΦPSII increases with higher R light levels and can also be elevated by moderate FR input, contributing to greater light use efficiency [51,57]. Although data are limited on EOD G light on Fv’/Fm’ and ΦPSII, G light penetrates deeper into the leaf and may support photosynthesis in internal tissues, potentially stabilizing or slightly improving these parameters under high irradiance.

In *C. officinalis*, Fv’/Fm’ values remained similar across treatments and gradually increased over time, reaching values near 0.8. This indicates a physiologically healthy and functional PSII, which in turn favors higher ΦPSII and supports greater ETR, as confirmed by the observed results.

### 4.3. Chlorophyll and Anthocyanin Responses to EOD Light Treatments

Under EOD R:FR treatment, Chl_Inx content per leaf area was noticeably reduced compared to plants exposed to G and control light. This is consistent with previous findings in *Arabidopsis* spp., where FR light reduced chlorophyll accumulation [58], and short-term EOD exposure to R:FR ratios similarly led to decreased leaf chlorophyll content [43]. Phytochrome-mediated shade signals can downregulate chlorophyll biosynthesis, likely as part of a resource reallocation strategy favoring elongation over pigmentation [59]. Although pigment composition was not analyzed, the overall reduction in chlorophyll under R:FR light suggests that *C. officinalis* partially activated a shade-avoidance process, prioritizing growth-related processes over photosynthetic pigment accumulation. In contrast, G and control lights retained higher total chlorophyll levels, indicative of a physiological state typical for high-light conditions [60]. G light suppresses shade-response pathways mediated by phytochromes, allowing sustained chlorophyll production under red/blue spectra [61], while control R:B light directly enhances chlorophyll biosynthesis and anthocyanin synthesis through phytochrome and cryptochrome activation [62].

Leaves accumulate low levels of anthocyanins in response to light and environmental cues, with visibly higher pigment levels under G and control lights. Under R:FR, however, anthocyanins were markedly reduced, indicating robust suppression of this pathway. Low R:FR ratios have been shown to inhibit anthocyanin production in various species, including *A. thaliana* [63], *L. sativa* [64], and *S. lycopersicum* varieties [65]. Mechanistically, FR light leads to phytochrome inactivation, destabilizing HY5 and downregulating late biosynthetic genes essential for anthocyanin accumulation [66]. In our study, EOD R:FR *C. officinalis* showed clear reductions in anthocyanin levels, whereas G light preserved their accumulation to high levels similar to those observed in *L. sativa* under G light [67]. G wavelengths are not strongly repressive to anthocyanin synthesis, and some evidence suggests they may attenuate shade-avoidance responses, indirectly allowing greater anthocyanin retention [68,69]. While B light is typically most effective in inducing anthocyanins, the G light treatment here functioned as a non-repressive spectral condition, enabling moderate pigment accumulation.

### 4.4. Biomass Responses to EOD Light Treatments

Despite exhibiting greater morphological measurements under EOD R:FR treatments, *C. officinalis* accumulated more biomass under EOD G 2 h treatment. This can be attributed to differences in growth strategy: R:FR light induces shade-avoidance responses, promoting elongation and expansion with low tissue density, while G light enhances light penetration within the canopy, sustaining photosynthesis in lower leaves and improving overall carbon gain [15]. G light has been shown to increase biomass in species like *S. lycopersicum* [70] and *O. basilicum* [71] by modifying plant architecture and promoting more efficient light distribution, leading to dense, productive growth despite its lower photosynthetic efficiency per unit leaf area.

### 4.5. Chemical Composition—FTIR of EOD Light Treatments

Infrared spectroscopic analyses revealed that the O–H and aromatic C=C bands-key signatures of phenolic compounds were most prominent in leaves exposed to control and EOD G light. This pattern indicates reduced levels of phenolic compounds, including flavonoids, under FR-enriched light. Such a reduction aligns with shade response, where low R:FR ratios downregulate flavonoid biosynthesis [72]. Conversely, G wavelengths do not strongly trigger shade-avoidance pathways, allowing for sustained flavonoid production [17]. Given that flavonol glycosides contribute to *C. officinalis*’ antioxidant properties, a reduction under EOD R:FR treatments may indicate a shift in resource allocation away from secondary metabolism under extended low R:FR conditions.

C–H asymmetric and symmetric stretching bands, associated with aliphatic chains and lipid content, were detected across most treatments. However, the control and EOD G treatments notably lacked the ~2849 cm^−1^ band, suggesting reduced lipids under these conditions. This corresponds with the ~2921 and ~2852 cm^−1^ bands described in [73] for long-chain aliphatics and with the ~2924 and ~2854 cm^−1^ bands assigned to alkanes in [74]. The authors of [75] corroborated these findings and further attributed these bands (~2925 and ~2859 cm^−1^) to CH_2_-rich structures within fatty acid esters, particularly those associated with bioactive compounds like faradiol esters and glycerol-based lipids.

The ester-related C=O stretching band (~1730–1745 cm^−1^) was prominent in nearly all treatments, except in EOD G 4 h leaves, implying a reduction in ester-containing compounds (e.g., cutin, methylated pectin) under this specific light condition. This is supported by the 1739 cm^−1^ band (assigned to saturated ketones [74]) and further explained in [75], where C=O bands around 1735–1740 cm^−1^ are linked to esters of bioactive triterpenoids and fatty acids with α-amylase inhibitory properties.

Similarly, the aromatic C=C stretching band (~1599–1633 cm^−1^), associated with conjugated double bonds and carboxylates, was consistently present, supporting the presence of aromatic phenolic structures, as also shown in [73] (~1605 cm^−1^) and by the 1627 cm^−1^ band linked to amine groups in [74]. In [75], a shoulder peak around 1568 cm^−1^, attributed to C=C stretching, was noted in unsaturated fatty acid esters, such as oleic and linoleic acids, both reported to contribute to enzyme inhibitory and oxidative stress modulation.

In the fingerprint region (1500–500 cm^−1^), flower samples exhibited more diverse spectral features. C–O vibrations linked to flavonoids and glycosides (~1049–1011 cm^−1^), as well as ester and glycosidic linkages (~1232–1144 cm^−1^), were detected exclusively in flowers. These bands coincide with the C–O–C stretching and glycosidic bond signals reported in [73]. In [75], bands in this range (~1070, 1150, 1230 cm^−1^) were attributed to esters, lactones, and faradiol derivatives with anti-inflammatory and anti-diabetic bioactivities, reinforcing the pharmacological relevance of these functional groups.

The peaks at 1064 and 1103 cm^−1^ confirmed the presence of aliphatic amines through C–N stretching, further supporting the complexity of nitrogen-containing metabolites in floral tissues [74]. Likewise, [75] described the diagnostic use of this region for distinguishing mono-, di- and triglycerides via O–CH_2_ vibrations (~1374 cm^−1^) and C–O–C ester signals, linking chemical structure with bioactivity through α-amylase inhibition assays.

Faradiol esters, saturated fatty acid esters like palmitic, lauric, and myristic acids, and phytoecdysteroids are strongly associated with α-amylase inhibitory activity in flower extracts [75]. These bands overlap with several functional groups identified in *C. officinalis’* FTIR spectra (e.g., C=O, C–O–C, and CH_2_-related bands), indicating that treatments that displayed such bands prominently in flowers may enhance bioactive accumulation.

### 4.6. Chemical Composition—GC-MS of EOD Light Treatments

Phytochemical analysis of hydromethanolic extracts from control leaves and flowers revealed terpenes and terpenoids (more abundant in leaves), lipophilic compounds such as fatty acids and their esters, phenolics, and nitrogenous heterocycles. Among terpenoids, aromadendrene, *α*-cadinol, and *β*-panasinsene were detected in leaves, while cedrene was found in flowers. The detection of α-cadinol—also reported by [28,76,77]—is particularly relevant due to its antimicrobial and antioxidant activities, and its inhibition of cholinesterase and tyrosinase enzymes. Both extracts contained 3-methyl-pentanoic acid but differed in the presence of 2-methylbutanoic acid (in leaves) and 3-methylbutanoic acid (in flowers). Regarding fatty acids, both extracts included methyl hexadecanoate and methyl stearate. Phenolics differed by position (2,6-dimethoxy in leaves and 3,4-dimethoxy in flowers). Nitrogenous heterocycles in leaves comprised pyrroles, imidazolidinone, and benzodiazepine derivatives, while indoles predominated in flowers.

Under EOD G 2 h, extracts contained sesquiterpenes, with flowers exhibiting a broader diversity of terpene skeletons, including eudesmane, cadinane, and selinane types. The lipid profile revealed a higher abundance of long-chain fatty acid esters in leaves, absent in flowers. Analogously, supplementation of hydroponic *L. sativa* with green light in combination with adequate nitrogen supply has been shown to raise ascorbic acid and other antioxidants [78], a trend mirrored in our leaves, which also exhibited more polycyclic nitrogen-containing heterocycles, whereas flowers contained simpler structures. Flowers were richer in oxygenated compounds, particularly those bearing carbonyl or hydroxyl groups.

Under EOD G 4 h treatments, leaves and flowers exhibited sesquiterpene-rich profiles with distinct distributions: longifolene and calacorene were more abundant in leaves, while eudesmol- and cubebol-type compounds predominated in flowers. Notably, calacorene reflects the synthesis of volatile sesquiterpenes with bioactive and ecological functions, contributing to the essential oil’s aromatic profile [29]. Cubebol showed affinity to tyrosinase, suggesting potential roles in pigmentation regulation and enzymatic browning processes [29]. Flowers also contained methyl esters of hexadecanoic and stearic acids. These compounds are notable due to their stability and role in maintaining membrane integrity in tissues exposed to oxidative stress [79]. Nitrogen-containing compounds were more diverse in leaves, including benzodiazepines and pyridine derivatives, whereas flowers contained carbazole-type structures. Leaves also presented butyrolactone among their lactones and cyclic carbonyls, while flowers featured 3-methyl-1,2-cyclopentanedione.

Under EOD R:FR 2 h treatment, leaves contained specific terpenoids such as isoaromadendrene epoxide and ledol, while flowers exhibited greater chemical diversity, including *β*-humulene, *γ*-eudesmol, and azulene-type compounds. Several of these, particularly ledol and *α*-humulene, exhibit dual activity as antioxidants and inhibitors of oxidative enzymes [28]. Methyl esters of stearic and pentadecanoic acids were detected in leaves, whereas flowers contained methyl hexadecanoate and tiglic acid. Both extracts exhibited complex nitrogenous heterocycles; however, leaves showed a higher prevalence of benzodiazepines, while flowers contained pyrimidine, pteridine, and sulfur–nitrogen compounds. In EOD R:FR 4 h treatments, despite shared sesquiterpenes and methyl esters, flowers displayed more oxygenated compounds, carboxylic acids, nitrogenous heterocycles, and unsaturated fatty acids—metabolites known for their bioactive properties [79].

Green light treatments, particularly EOD G 4 h in leaves, induced the synthesis of different terpenes compared to control leaves, including *α*-pinene, longifolene, *α*-selinene, cadalene, and megastigmatrienone, as well as esters (e.g., tiglic acid), and lactones and ketones (e.g., butyrolactone, hydroxyacetone, and 2-hydroxy-3-methyl-2-cyclopenten-1-one). In parallel, flowers revealed elevated levels of aromatic sesquiterpenes (e.g., ylangene, aromadendrene), aldehydes (e.g., 4-propylbenzaldehyde diethyl acetal), and acids such as tiglic acid under green light. Comparable metabolites have been reported in recent studies showing that green light modulates the expression of terpene synthase genes and the biosynthetic pathways involved in volatile production [80]. The potential of green light to steer terpenoid composition and enhance the accumulation of sesquiterpenes and monoterpenes has also been highlighted in broader reviews of light-driven metabolite regulation [81]. Additionally, experiments in lettuce seedlings showed that green light influences leaf metabolism and secondary compound dynamics, consistent with the observed induction of esters and ketones in this study [82].

Comparison of EOD R:FR 4 h and the control leaves extracts indicated that R:FR light altered the phytochemical profile exhibiting reduced chemical diversity, changes in sesquiterpene composition (emergence of *β*-humulene and loss of aromadendrene, *α*-calacorene, cedrene-V6, and other terpenes present in the control leaves), and an increase in nitrogen-containing heterocycles, including 4-phenyl-pyrido[2,3-d]pyrimidine, 1-(4-nitrophenyl)piperazine, and 2,4-dimethyl-benzo[h]quinoline. Modified amino acids (e.g., N-ethoxycarbonyl-D-alanine, hexyl ester) were also detected, along with a reduction in small organic acids such as 2-methylhexanoic and tiglic acids. These changes suggest that R:FR light modulates secondary metabolism, promoting the biosynthesis of nitrogenous heterocycles and altering sesquiterpene composition, likely through differential regulation of the mevalonate and methylerythritol phosphate pathways [83,84,85].

The comparison between EOD R:FR 4 h and the control flower extracts confirmed that R:FR light induced broader changes in floral phytochemistry. Flowers under R:FR contained unsaturated fatty acids (*α*-linolenic and linoleic acids), a greater variety of nitrogenous heterocycles (e.g., 5,6-dimethyl-1,10-phenanthroline, 2-methyl-6-(5-methyl-2-thiazolin-2-ylamino)pyridine, purine-2,6-dione, 2-ethylacridine, 2-methylpiperazine, and pyrido[3,2-d]pyrimidin-4(3H)-one), and specific terpenes (e.g., cadala-1(10),3,8-triene, *γ*-eudesmol, and 8,9-dehydro-cycloisolongifolene). Distinct phenolic compounds were also identified, including 2,5-bis(1,1-dimethylethyl)-1,4-benzenediol and syringol. R:FR light markedly influenced secondary metabolism in flowers, enhancing the production of polyunsaturated fatty acids, nitrogenous heterocycles, and diterpenes, while also modifying phenylpropanoid pathways. The presence of *γ*-eudesmol and methyl *α*-linolenate suggests potential activation of stress response and signaling mechanisms, whereas the reduction in indole derivatives may reflect shifts in tryptophan metabolism [79].

### 4.7. Applicability, Limitations, and Future Research

#### 4.7.1. Practical Applicability of Findings

The present findings demonstrate significant practical applications for the commercial cultivation of *C. officinalis* in controlled environments. The identification of EOD G 2 h as the optimal light treatment for enhancing biomass and phytochemical diversity offers a strategy for pharmaceutical and nutraceutical industries seeking to maximize production and bioactive compound yield. This is especially relevant given the increasing demand for standardized medicinal plant materials with consistent phytochemical profiles.

From an economic perspective, the implementation of EOD G treatments could reduce energy consumption compared to continuous lighting regimes, as the strategic application of specific wavelengths for short periods proved more effective than extended photostimulation. The observed increase in biomass under EOD G 2 h, coupled with enhanced accumulation of bioactive compounds such as *α*-cadinol, represents a dual benefit for commercial producers—higher yield and superior product quality with lower costs.

For vertical farming and controlled environment agriculture systems, these findings offer a practical framework for spectral recipe development. The demonstrated feasibility of modulating secondary metabolite profiles through manipulating EOD light quality could enable producers to “customize” the phytochemical output based on market demands. Specifically, the modulation of sesquiterpene and flavonoid content through selective light application could allow for the targeted enhancement of specific compounds with established therapeutic value.

#### 4.7.2. Limitations of the Study

Despite the valuable insights provided by this investigation, several limitations must be acknowledged. First, the study focused exclusively on controlled hydroponic conditions, which limits direct extrapolation to field-grown *C. officinalis* or plants cultivated under different substrate systems. Interactions between light and edaphic factors affecting metabolite accumulation remain unexplored.

The research was conducted over a single growth cycle, preventing the assessment of seasonal effects or long-term physiological adaptations to EOD treatments. Additionally, the photon flux density remained constant for all EOD treatments, precluding analysis of potential interactions between light intensity and spectral quality. Light intensity is known to influence photomorphogenic responses and could potentially modulate the effects observed in this study.

Another limitation lies in the analytical approach to phytochemical profiling. While GC-MS provided insight data on volatile and semi-volatile compounds, complementary techniques such as high-performance liquid chromatography coupled with mass spectrometry (HPLC-MS) or nuclear magnetic resonance spectroscopy could better characterize polar compounds, including flavonoid glycosides, which contribute significantly to the medicinal properties of *C. officinalis*.

Finally, the study did not include an assessment of the biological activities of the extracts derived from plants grown under different light treatments. In particular, antioxidant capacity was not directly evaluated through widely used methods such as the 2,2-diphenyl-1-picrylhydrazyl radical scavenging assay (DPPH), the ferric reducing antioxidant power assay (FRAP), or the 2,2’-azino-bis(3-ethylbenzothiazoline-6-sulfonic acid) radical cation decolorization assay (ABTS). Correlating phytochemical profiles with therapeutic potential through bioassays (e.g., antioxidant, anti-inflammatory, or antimicrobial activities) would provide more explicit evidence regarding the functional significance of the observed metabolic shifts.

#### 4.7.3. Future Research Directions

Several promising avenues for future research emerge from this investigation. First, expanding the spectral manipulation approaches to include combinations of different wavelengths, pulse frequencies, and varied photon flux densities could yield more refined lighting strategies. In particular, investigating the effects of pulsed EOD treatments with varying duty cycles may enhance photosynthetic efficiency and reduce energy use.

To deepen the understanding of phytochemical modulation, future studies should consider performing two complementary extraction protocols. For the volatile profile, hydrodistillation using a Clevenger apparatus is recommended, as it allows efficient recovery of essential oil components while minimizing the loss of thermolabile volatiles—thus providing a more accurate characterization of light-induced shifts in secondary metabolism. In parallel, a second extraction using polar solvents such as methanol or hydro-methanol, as applied in the present study, would permit the analysis of non-lipophilic compounds, including phenolics. This strategy would broaden the metabolite coverage and facilitate a more comprehensive chemical profile. Subsequent analysis using high-performance liquid chromatography coupled with a diode array detector (HPLC-DAD) is advised to improve resolution and quantification of key phenolic constituents.

Detailed transcriptomic and metabolomic analyses would provide deeper insights into the molecular mechanisms underlying phytochemical shifts. Identifying key regulatory genes and metabolic pathways responsive to specific spectral cues could facilitate targeted genetic approaches to enhance desirable traits in *C. officinalis* and potentially other medicinal species.

Field trials incorporating EOD light supplementation in greenhouse or polytunnel systems would bridge the gap between controlled environment findings and practical horticultural applications. Such studies would help determine whether the benefits observed under highly controlled conditions persist in more variable environments.

Long-term studies spanning multiple growth cycles would reveal potential cumulative or adaptive responses to EOD treatments, which could influence production strategies for perennial cultivation systems. Additionally, examining the effects of EOD treatments on seed quality and subsequent generation performance would be valuable for breeding programs focused on enhanced phytochemical production.

Future work should also include bioactivity assays of extracts from plants grown under different light treatments. Correlating phytochemical profiles with therapeutic effects would directly support the functional relevance of metabolic changes and guide the development of light-based strategies for enhancing specific bioactive compounds.

Finally, economic and environmental impact assessments of implementing EOD lighting strategies at commercial scale would provide crucial information for industry adoption. Analysis of energy consumption, installation costs, and return on investment through enhanced crop value would help establish the practical viability of these techniques for sustainable production of high-quality medicinal plant materials.

## 5. Conclusions

*Calendula officinalis* plants exposed to R:B light supplemented with EOD G 2 h exhibited the most favorable physiological and productive responses, maintaining high chlorophyll and anthocyanin levels, and promoting the highest biomass accumulation. It also preserved elevated levels of phenolic compounds in leaves—metabolites often associated with antioxidant potential—and induced a diverse array of terpenoid skeletons in flowers, particularly eudesmanes, cadinanes, and selinanes. In contrast, EOD R:FR triggered shade-avoidance elongation without increasing biomass, while reducing pigment and phenolic accumulation. The EOD R:FR 2 h improved water-use efficiency, whereas the 4 h variant showed signs of photosynthetic imbalance. The control treatment supported phenolic accumulation and stable physiological function.

Phytochemicals were more abundant in flowers than in leaves, particularly nitrogen-containing compounds and oxygenated terpenes. This is consistent with their defense and reproduction roles. However, a consistent metabolic baseline across treatments was indicated by the similar core group of metabolites found in all the extracts, including carboxylic acids and the sesquiterpene α-cadinol. The findings show that EOD-G light is a promising method for increasing *Calendula officinalis* biomass and phytochemical yields, which is helpful for controlled-environment farming and the production of medicinal plants.

## Figures and Tables

**Table 1:** Results of two-way analysis of variance (ANOVA) for morphological and reproductive variables of *C. officinalis* evaluated at 27, 42, 56, 70, and 84 days after sowing (DAS) under different light treatments. *p*-values ≤ 0.05 indicate statistically significant effects.

Variable	*p*-Value (Treatment)	*p*-Value (Time, DAS)	*p*-Value (Treatment × Time)
Leaf number	0.0003	<0.0001	0.0087
Stem length	<0.0001	<0.0001	<0.0001
Leaf width	0.0001	<0.0001	<0.0001
Leaf length	<0.0001	<0.0001	<0.0001
Flower bud number	<0.0001	<0.0001	0.0019
Flower number	<0.0001	<0.0001	<0.0001
Floret length	0.0001	<0.0001	0.0336
Capitulum diameter	<0.0001	<0.0001	0.0284
Floral stem length	<0.0001	<0.0001	0.0001

**Table 2:** Results of two-way ANOVA for gas exchange and chlorophyll fluorescence variables of *C. officinalis* evaluated at 27, 42, 56, 70, and 84 DAS under different light treatments.

Variable	*p*-Value (Treatment)	*p*-Value (Time, DAS)	*p*-Value (Treatment × Time)
A	0.0409	<0.0001	0.0037
E	<0.0001	0.0004	0.0143
gsw	<0.0001	0.0030	0.1785
ΦPSII	<0.0001	0.0071	0.0051
ETR	<0.0001	<0.0001	0.0156
Fv’/Fm’	0.0004	<0.0001	0.0018

*p*-values ≤ 0.05 indicate statistically significant effects; A, E, gsw, ΦPSII, ETR, and Fv’/Fm’ stand for assimilation rate, transpiration rate, stomatal conductance to water vapor, electron transport rate, effective quantum yield of photosystem II, and maximum quantum efficiency of photosystem II in the light-adapted state, respectively.

**Table 3:** Chlorophyll index (Chl-Idx) of *C. officinalis* evaluated at 27, 42, 56, 70, and 84 DAS under different light treatments.

Treatment	27 DAS	42 DAS	56 DAS	70 DAS	84 DAS
Control	0.70 ± 0.10 ^a^	1.09 ± 0.25 ^a^	1.12 ± 0.26 ^b^	1.11 ± 0.25 ^a^	0.73 ± 0.24 ^a^
EOD G 4 h	0.76 ± 0.11 ^a^	0.97 ± 0.22 ^a^	0.92 ± 0.26 ^a^	1.09 ± 0.27 ^a^	0.81 ± 0.27 ^b^
EOD G 2 h	0.81 ± 0.12 ^a^	1.05 ± 0.26 ^a^	1.01 ± 0.25 ^a^	1.16 ± 0.34 ^b^	1.08 ± 0.34 ^b^
EOD R:FR 2 h	0.74 ± 0.12 ^a^	0.85 ± 0.17 ^a^	0.69 ± 0.15 ^a^	0.82 ± 0.26 ^a^	0.97 ± 0.30 ^a^
EOD R:FR 4 h	0.79 ± 0.14 ^a^	0.64 ± 0.14 ^b^	0.76 ± 0.16 ^a^	0.81 ± 0.23 ^b^	0.96 ± 0.25 ^b^

Chl-Idx values are expressed as mean ± standard deviation. Values in the same column followed by the same letter indicate no significant differences according to Tukey’s HSD test (α = 0.05).

**Table 4:** Anthocyanin index (Ari_Idx) of *C. officinalis* evaluated at 27, 42, 56, 70, and 84 DAS under different light treatments.

Treatment	27 DAS	42 DAS	56 DAS	70 DAS	84 DAS
Control	1.90 ± 0.52 ^a^	3.62 ± 2.61 ^a^	3.66 ± 1.55 ^a^	3.34 ± 1.37 ^a^	2.48 ± 1.15 ^a^
EOD G 4 h	1.85 ± 0.50 ^a^	3.01 ± 1.25 ^a^	2.94 ± 1.26 ^a^	3.67 ± 1.60 ^a^	2.69 ± 1.37 ^a^
EOD G 2 h	1.99 ± 0.57 ^a^	3.44 ± 1.67 ^a^	3.19 ± 1.35 ^a^	3.92 ± 1.60 ^a^	3.71 ± 1.73 ^a^
EOD R:FR 2 h	1.70 ± 0.50 ^a^	2.66 ± 1.02 ^b^	1.97 ± 0.66 ^b^	2.47 ± 1.23 ^b^	3.26 ± 1.61 ^a^
EOD R:FR 4 h	1.91 ± 0.67 ^a^	1.89 ± 0.66 ^c^	2.22 ± 0.80 ^b^	2.93 ± 1.30 ^a^	3.21 ± 1.37 ^a^

Ari_Idx values are expressed as mean ± standard deviation. Values in the same column followed by the same letter indicate no significant differences according to Tukey’s HSD test (α = 0.05).

**Table 5:** ANOVA for fresh and dry biomass of *C. officinalis* under different light treatments.

Treatment	Fresh Weight				
	Leaf	Stem	Bud	Flower	Total
Control	201.47 ± 8.28 ^a^	217.47 ± 14.64 ^b^	6.65 ± 0.54 ^a^	11.29 ± 0.39 ^a^	436.88
EOD G 4 h	201.1 ± 26.33 ^a^	237.42 ± 28.1 ^a^	4.14 ± 1.45 ^a^	9.29 ± 0.69 ^a^	451.95
EOD G 2 h	286.22 ± 31.12 ^a^	287.3 ± 17.96 ^a^	7.54 ± 0.46 ^a^	19.81 ± 6.04 ^a^	600.86
EOD R:FR 4 h	256.64 ± 29.31 ^a^	294.9 ± 24.31 ^a^	5.05 ± 0.92 ^a^	9.23 ± 4.27 ^a^	565.81
EOD R:FR 2 h	245.46 ± 22.84 ^a^	362.54 ± 26.65 ^c^	7.43 ± 1.53 ^a^	14.21 ± 2.89 ^a^	629.64
Pr > F	0.144	0.009	0.176	0.264	-
	** Dry weight **				
Control	18.11 ± 0.72 ^b^	20.29 ± 1.53 ^b^	0.9 ± 0.17 ^a^	2.24 ± 0.17 ^a^	41.54
EOD G 4 h	14.74 ± 1.82 ^b^	16.38 ± 1.15 ^b^	0.78 ± 0.06 ^a^	1.69 ± 0.28 ^a^	33.58
EOD G 2 h	27.61 ± 3.18 ^a^	28.5 ± 4.81 ^a^	1.14 ± 0.08 ^a^	3.91 ± 1.06 ^a^	61.16
EOD R:FR 4 h	20.28 ± 2.28 ^a^	22.66 ± 2.53 ^b^	0.77 ± 0.18 ^a^	1.22 ± 0.49 ^a^	44.93
EOD R:FR 2 h	20.97 ± 2.03 ^a^	31.1 ± 3.53 ^a^	1.06 ± 0.19 ^a^	2.48 ± 0.4 ^a^	55.61
Pr > F	0.019	0.035	0.344	0.062	-

Data represent means ± SEM. Values in the same column followed by the same letter indicate no significant differences according to Tukey’s HSD test (α = 0.05).

**Table 6:** Main bands in the infrared spectra of *C. officinalis* leaf (L) and flower (F) hydromethanolic extract under different light treatments.

Control		EOD G 4 h		EOD G 2 h		EOD R:FR 4 h		EOD R:FR 2 h		Assignment
L	F	L	F	L	F	L	F	L	F	
3294	3391	3282		3361		3393		3342	3346	O–H stretching (hydrogen-bonded hydroxyls, polyphenols, water)
2917	2918	2917	2917	2917	2918	2917	2917	2917	2917	C–H asymmetric and symmetric stretching (aliphatic chains, lipids, waxes)
	2849		2849		2850	2849	2849	2849	2849	C–H asymmetric and symmetric stretching (aliphatic chains, lipids, waxes)
							2156			Strong C=C=O ketone (stretching)
1742	1734		1735	1745	1734	1739	1734	1739	1733	C=O stretching (esters in cutin, methylated pectin, fatty acids)
1633	1612	1599	1607	1620	1610	1614	1612	1612	1604	C=C stretching (aromatic rings, conjugated alkenes, carboxylates)
	1462		1462				1463			C–H bending (–CH_2_ scissoring deformation in aliphatic chains; cuticular wax/cutin)
1322		1331		1324		1336		1352		CH_2_ and CH_3_ bending, O–H in-plane bending (lignin, polyphenols)
	1233		1262		1237		1236		1232	Strong C–O–C and a carboxylic ester
	1144								1146	C–O–C stretching (asymmetric stretch of ester linkages in cutin; also characteristic of glycosidic bonds)
1104	1105	1104	1106	1104	1104	1103	1105	1102		C–O–C and C–O stretching (polysaccharides, glycosidic bonds, esters)
	1049				1050				1048	C–O vibration (flavones or terpenoids)
	1012		1011				1011			C–O stretching (glycosidic linkages in polysaccharides or glycosides)
957	976		962				962			Out-of-plane C–H bending (aromatics, trans alkenes)
904	900		913	913	916	909	913			Out-of-plane C–H bending (aromatics, trans alkenes)
		825		825	819	825		824		Out-of-plane C–H bending (aromatics, trans alkenes)
	720		720				720			Stretching vibration of CH_2_
667				635	626	669		638		Ring deformation, C–H wagging, possible C–X bending
537	537	534	490	535	534		534		534	Ring deformation, C–H wagging, possible C–X bending
		423		423						Skeletal deformation, out-of-plane ring torsion

Wavenumber values are expressed in cm^−1^.

## Data Availability

Data are contained within the article and Supplementary Materials.

## Supplementary Materials

The following supporting information can be downloaded at: https://www.mdpi.com/article/10.3390/biology14080935/s1, Figure S1: Measured spectral profiles of light treatments: R:B, G, and R:FR; Figures S2 and S3: FTIR spectra of *C. officinalis* leaf and flower hydromethanolic extracts under different end-of-day light treatments; Figures S4–S8: GC–MS chromatograms of *C. officinalis* leaf hydromethanolic extracts under different treatments (control, EOD G 2 h, EOD G 4 h, EOD R:FR 2 h, and EOD R:FR 4 h); Figures S9–S13: GC–MS chromatograms of *C. officinalis* flower hydromethanolic extracts under different treatments (control, EOD G 2 h, EOD G 4 h, EOD R:FR 2 h, and EOD R:FR 4 h); Tables S1 and S2: Chlorophyll and Anthocyanin indices and mean distribution of leaf area (%) across five classes in *C. officinalis* evaluated at different days after sowing under different end-of-day light treatments; Tables S3–S7: Main chemical compounds identified by GC-MS in *C. officinalis* leaf extracts under different treatments; Tables S8–S12: Main chemical compounds identified by GC-MS in *C. officinalis* flower extracts under different treatments.

## Abbreviations

The following abbreviations are used in this manuscript:

A	Net photosynthetic assimilation rate
Ari_Idx	Anthocyanin index
ATR	Attenuated total reflectance
B	Blue
Chl-Idx	Chlorophyll index
DAS	Days after sowing
E	Transpiration rate
EOD	End-of-day
ETR	Electron transport rate
FR	Far-red
FTIR	Fourier transform infrared
Fv’/Fm’	Maximum quantum efficiency of PSII in light-adapted state
G	Green
GC-MS	Gas chromatography-mass spectrometry
gsw	Stomatal conductance to water vapor
HPLC	High-performance liquid chromatography
LDP	Long-day plant
PPFD	Photosynthetic photon flux density
PSI	Photosystem I
PSII	Photosystem II
R	Red
SEM	Standard error of the mean
UV-A	Ultraviolet A
UV-B	Ultraviolet B
UVR8	UV resistance locus 8
ΦPSII	Effective quantum yield of PSII
