# Supplementary material for: Physiological and Phytochemical Responses of Calendula officinalis L. to End-of-Day Red/Far-Red and Green Light
Source: Biology (Basel). 2025 Jul 24;14(8):935. doi: 10.3390/biology14080935 (PMC12383836; doi:10.3390/biology14080935)
Supplement: Supplementary file 1 [file biology-14-00935-s001.zip › biology-3721119-supplementary.pdf]

Physiological and Phytochemical Responses of *Calendula officinalis L.* to End-of-Day Red/Far-Red and Green Light Spectral Treatments

Luisa F. Lozano-Castellanos, Giuseppina Pennisi, Luis Manuel Navas-Gracia, Francesco Orsini, Eva Sán-chez-Hernández, Pablo Martín-Ramos, and Adriana Correa-Guimaraes

SUPPORTING INFORMATION

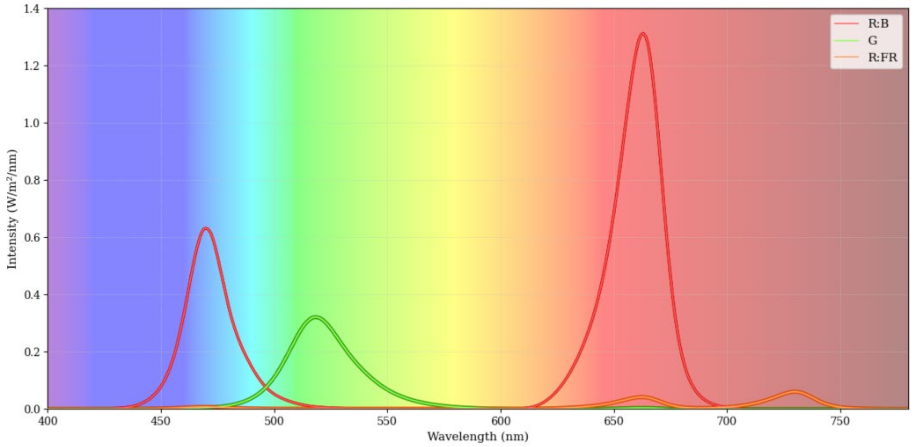

Figure S1. Measured spectral profiles of light treatments: R:B, G, and R:FR.

Table S1. Chlorophyll index (Chl-Idx) and mean distribution of leaf area (%) across five Chl-Idx classes in *Calendula officinalis* L. evaluated at 27, 42, 56, 70, and 84 days after sowing (DAS) under different end-of-day light treatments.

DAS	Treatment	Chl-Idx	Class I (%)	Class II (%)	Class III (%)	Class IV (%)	Class V (%)
27	Control	0.70 ± 0.10	0.2	13.2	82.3	0	0
	EOD G 4h	0.76 ± 0.11	0.5	14.7	82.1	0.9	0
	EOD G 2h	0.81 ± 0.12	0.1	8.3	84.6	5.2	0
	EOD R:FR 2h	0.74 ± 0.12	0.7	19.9	76.7	1.0	0
	EOD R:FR 4h	0.79 ± 0.14	0.7	13.7	79.5	4.4	0.1
42	Control	1.09 ± 0.25	0.5	3.4	30.7	49.1	12.3
	EOD G 4h	0.97 ± 0.22	0.9	5.3	48.7	38.1	4.8
	EOD G 2h	1.05 ± 0.26	0.1	4.6	41.6	39.1	10
	EOD R:FR 2h	0.85 ± 0.17	0.7	10.8	68.9	16.3	1.4
	EOD R:FR 4h	0.64 ± 0.14	2.0	55.4	39.9	1.0	0
56	Control	1.12 ± 0.26	0.3	3.3	27.2	50	14.6
	EOD G 4h	0.92 ± 0.26	3.6	8.2	51.9	30.3	3.6
	EOD G 2h	1.01 ± 0.25	1.3	5.5	42.0	40.0	8.0
	EOD R:FR 2h	0.69 ± 0.15	0.7	43.3	52.8	1.6	0
	EOD R:FR 4h	0.76 ± 0.16	0.5	25.4	65.3	7.1	0.2
70	Control	1.11 ± 0.25	0.5	3.7	27.2	50.8	14.5
	EOD G 4h	1.09 ± 0.27	0.8	4.7	28.5	50.3	10.6
	EOD G 2h	1.16 ± 0.34	4.0	3.2	13.6	51.7	21.6
	EOD R:FR 2h	0.82 ± 0.26	3.4	21.3	51.2	19.9	2.3
	EOD R:FR 4h	0.81 ± 0.23	4.5	16.3	60.6	16.3	0.8
84	Control	0.73 ± 0.24	4.5	36.2	44.7	12.2	0.6
	EOD G 4h	0.81 ± 0.27	5.1	21.7	50.3	18.8	2.1
	EOD G 2h	1.08 ± 0.34	2.8	8.2	25.0	41.5	17.5
	EOD R:FR 2h	0.97 ± 0.30	3.0	11.2	37.9	36.2	8.6
	EOD R:FR 4h	0.96 ± 0.25	2.5	8.5	41.3	41.1	4.4

Chl-Idx values are expressed as mean ± standard deviation. Class I (0.00–0.30), Class II (0.31–0.60), Class III (0.61–0.90), Class IV (0.91–1.20), and Class V (1.21–1.50).

Table S2. Anthocyanin index (Ari_Idx) and mean distribution of leaf area (%) across five Ari_Idx classes in *C. officinalis* evaluated at 27, 42, 56, 70, and 84 DAS under different light treatments.

DAS	Treatment	Ari_Idx (± SD)	Class I (%)	Class II (%)	Class III (%)	Class IV (%)	Class V (%)
27 DAS	Control	1.90 ± 0.52	62.2	36.2	0.2	0.0	0.0
	EOD G 4h	1.85 ± 0.50	66.8	31.6	0.2	0.0	0.0
	EOD G 2h	1.99 ± 0.57	56.6	41.4	0.5	0.0	0.0
	EOD R:FR 2h	1.70 ± 0.50	80.8	17.6	0.2	0.0	0.0
	EOD R:FR 4h	1.91 ± 0.67	60.6	37.5	0.6	0.1	0.0
42 DAS	Control	3.62 ± 2.61	9.1	59.0	23.1	5.5	1.3
	EOD G 4h	3.01 ± 1.25	16.6	65.0	14.7	2.1	0.3
	EOD G 2h	3.44 ± 1.67	14.7	58.2	18.0	5.0	1.6
	EOD R:FR 2h	2.66 ± 1.02	21.7	69.3	6.7	1.0	0.2
	EOD R:FR 4h	1.89 ± 0.66	62.8	35.3	0.7	0.0	0.0
56 DAS	Control	3.66 ± 1.55	10.4	56.9	24.1	5.4	1.6
	EOD G 4h	2.94 ± 1.26	20.9	63.2	12.7	1.7	0.3
	EOD G 2h	3.19 ± 1.35	15.4	63.4	16.6	2.7	0.7
	EOD R:FR 2h	1.97 ± 0.66	55.9	42.5	0.8	0.0	0.0
	EOD R:FR 4h	2.22 ± 0.80	40.7	55.0	3.1	0.0	0.0
70 DAS	Control	3.34 ± 1.37	15.8	56.9	22.4	3.5	0.4
	EOD G 4h	3.67 ± 1.60	8.7	60.1	22.6	5.0	1.5
	EOD G 2h	3.92 ± 1.60	9.8	46.2	34.3	6.6	1.3
	EOD R:FR 2h	2.47 ± 1.23	37.1	52.5	7.8	1.3	0.2
	EOD R:FR 4h	2.93 ± 1.30	21.3	62.9	13.1	1.4	0.2
84 DAS	Control	2.48 ± 1.15	37.4	52.2	8.6	0.8	0.1
	EOD G 4h	2.69 ± 1.37	30.9	54.3	11.4	1.8	0.3
	EOD G 2h	3.71 ± 1.73	15.8	43.2	31.0	6.7	1.3
	EOD R:FR 2h	3.26 ± 1.61	21.5	51.0	20.5	4.4	0.9
	EOD R:FR 4h	3.21 ± 1.37	17.4	59.4	18.4	2.9	0.4

Ari_Idx values are expressed as mean ± standard deviation. Class I (0.00–2.00), Class II (2.01–4.00), Class III (4.01–6.00), Class IV (6.01–8.00), and Class V (8.01–10.00).

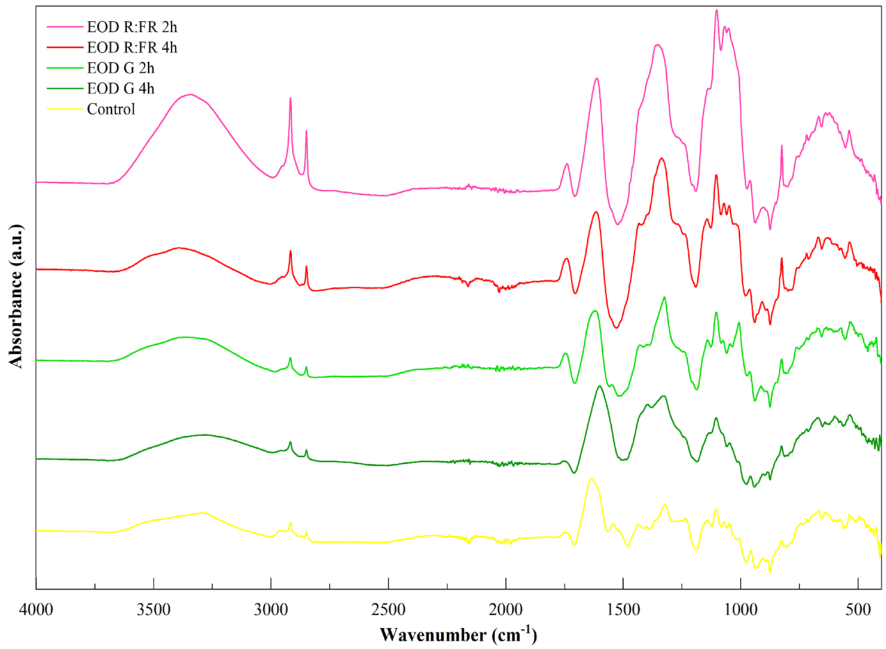

Figure S2. FTIR spectra of *C. officinalis* leaf hydromethanolic extract under different end-of-day light treatments.

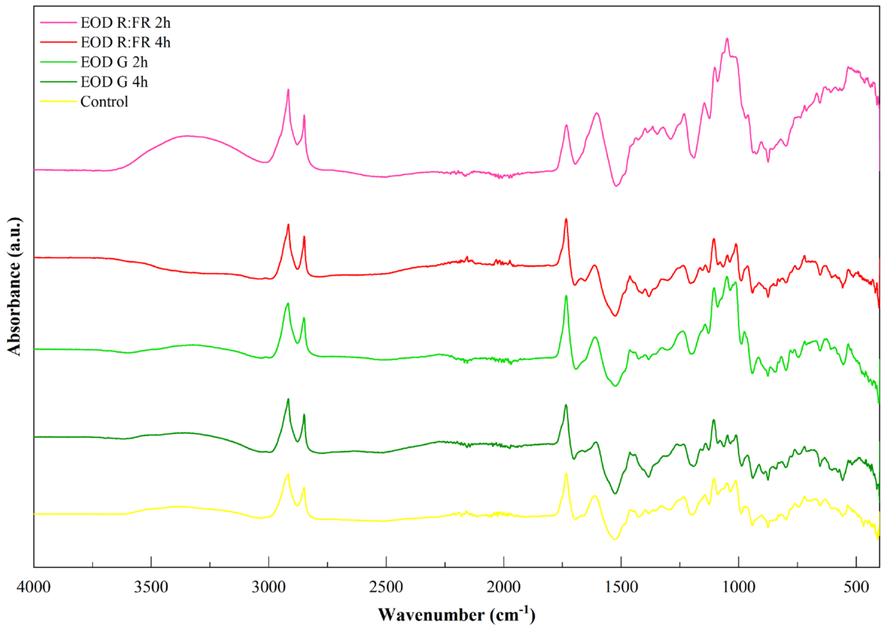

Figure S3. FTIR spectra of *C. officinalis* flower hydromethanolic extract under different end-of-day light treatments.

Table S3. Main chemical compounds identified by GC-MS in *C officinalis* leaf extract under the control treatment.

RT (min)	Area (%)	Assignment	Qual
5.1168	51.3115	Oxime-, methoxy-phenyl-_	91
15.3730	9.5541	α-Cadinol	99
13.0819	4.7398	Alloaromadendrene	99
13.4915	4.5189	1H-Cycloprop[e]azulene, 1a,2,3,5,6,7,7a,7b-octahydro-1,1,4,7-tetramethyl-, [1aR-(1aα,7α,7aβ,7bα)]-	98
6.2801	3.9713	7H-Dibenzo[b,g]carbazole, 7-methyl-	53
5.8705	3.0771	4-Imidazolidinone, 2-thioxo-	38
19.9788	2.0496	Methyl stearate	99
18.0676	2.0163	Hexadecanoic acid, methyl ester	98
5.4848	1.8120	2-Anthracenamine	38
5.6628	1.7193	Pentanoic acid, 3-methyl-	49
14.6964	1.4091	Ledol	99
13.7942	1.3323	Naphthalene, 1,2,3,5,6,8a-hexahydro-4,7-dimethyl-1-(1-methylethyl)-, (1S-cis)-	99
13.9841	1.2341	β-Guaiene	81
11.0402	1.0757	2-Methoxy-4-vinylphenol	60
9.6513	1.0414	Propionic acid, 3-(m-aminobenzoyl)-2-methyl-	58
12.8089	0.9871	Aromandendrene	87
8.2981	0.9365	4-Ethylbenzoic acid, 2-butyl ester	35
4.4223	0.7832	Butanoic acid, 3-methyl-	50
13.2244	0.7133	Cedrene-V6	44
15.7528	0.7068	p-Heptyloxyaniline	27
8.1853	0.6495	1,2,3-Propatriol, 1-indol-4-yl(ether)	47
15.5510	0.5939	Valeric acid, 2,6-dimethylnon-1-en-3-yn-5-yl ester	43
14.8744	0.5452	3-Buten-2-one, 4-(2,6,6-trimethyl-1-cyclohexen-1-yl)-	78
3.3184	0.4983	2-Propanone, 1-hydroxy-	45
16.3167	0.4720	N-Methyl-1-adamantaneacetamide	35
14.4589	0.4501	Methyl 3-(1-pyrrolo)thiophene-2-carboxylate	27
4.5232	0.3304	Butanoic acid, 2-methyl-	25
15.0643	0.3218	1H-Indole, 5-methyl-2-phenyl-	22
14.0672	0.3037	Quinoline, 5,8-dimethyl-	53
11.5447	0.2887	3-Amino-2,6-dimethoxypyridine	46
15.0228	0.2681	Cytisine	27
12.1264	0.1994	8-Methyl-13,14-dioxo-8H,13H,14H-naphtho(1',2':5,6)pyrano(2,3-b)quinoline	27
24.7982	0.0895	2-(Acetoxymethyl)-3-(methoxycarbonyl)biphenylene	47

Table S4. Main chemical compounds identified by GC-MS in *C. officinalis* leaf extract under EOD G 2h treatment.

RT (min)	Area (%)	Assignment	Qual
5.1168	51.3115	Oxime-, methoxy-phenyl-_	91
15.3730	9.5541	α-Cadinol	99
13.0819	4.7398	Alloaromadendrene	99
13.4915	4.5189	1H-Cycloprop[e]azulene, 1a,2,3,5,6,7,7a,7b-octahydro-1,1,4,7-tetramethyl-, [1aR-(1aα,7α,7aβ,7bα)]-	98
6.2801	3.9713	7H-Dibenzo[b,g]carbazole, 7-methyl-	53
5.8705	3.0771	4-Imidazolidinone, 2-thioxo-	38
19.9788	2.0496	Methyl stearate	99
18.0676	2.0163	Hexadecanoic acid, methyl ester	98
5.4848	1.8120	2-Anthracenamine	38
5.6628	1.7193	Pentanoic acid, 3-methyl-	49
14.6964	1.4091	Ledol	99
13.7942	1.3323	Naphthalene, 1,2,3,5,6,8a-hexahydro-4,7-dimethyl-1-(1-methylethyl)-, (1S-cis)-	99
13.9841	1.2341	β-Guaiene	81
11.0402	1.0757	2-Methoxy-4-vinylphenol	60
9.6513	1.0414	Propionic acid, 3-(m-aminobenzoyl)-2-methyl-	58
12.8089	0.9871	Aromandendrene	87
8.2981	0.9365	4-Ethylbenzoic acid, 2-butyl ester	35
4.4223	0.7832	Butanoic acid, 3-methyl-	50
13.2244	0.7133	Cedrene-V6	44
15.7528	0.7068	p-Heptyloxyaniline	27
8.1853	0.6495	1,2,3-Propatriol, 1-indol-4-yl(ether)	47
15.5510	0.5939	Valeric acid, 2,6-dimethylnon-1-en-3-yn-5-yl ester	43
14.8744	0.5452	3-Buten-2-one, 4-(2,6,6-trimethyl-1-cyclohexen-1-yl)-	78
3.3184	0.4983	2-Propanone, 1-hydroxy-	45
16.3167	0.4720	N-Methyl-1-adamantaneacetamide	35
14.4589	0.4501	Methyl 3-(1-pyrrolo)thiophene-2-carboxylate	27
4.5232	0.3304	Butanoic acid, 2-methyl-	25
15.0643	0.3218	1H-Indole, 5-methyl-2-phenyl-	22
14.0672	0.3037	Quinoline, 5,8-dimethyl-	53
11.5447	0.2887	3-Amino-2,6-dimethoxypyridine	46
15.0228	0.2681	Cytisine	27
12.1264	0.1994	8-Methyl-13,14-dioxo-8H,13H,14H-naphtho(1',2':5,6)pyrano(2,3-b)quinoline	27
24.7982	0.0895	2-(Acetoxymethyl)-3-(methoxycarbonyl)biphenylene	47

Table S5. Main chemical compounds identified by GC-MS in *C. officinalis* leaf extract under EOD G 4h treatment.

RT (min)	Area (%)	Assignment	Qual
5.1465	31.6413	Oxime-, methoxy-phenyl-_	91
15.3671	11.0004	α-Cadinol	99
13.4916	5.3937	1H-Cycloprop[e]azulene, 1a,2,3,5,6,7,7a,7b-octahydro-1,1,4,7-tetramethyl-, [1aR-(1aα,7α,7aβ,7bα)]-	96
15.2188	3.8883	Bicyclo[4.4.0]dec-1-ene, 2-isopropyl-5-methyl-9-methylene-	94
3.3838	3.2269	2-Propanone, 1-hydroxy-	59
8.2922	2.7279	Phthalic acid, 2,7-dimethyloct-7-en-5-yn-4-yl ethyl ester	43
5.8944	2.6644	3H-1,2,4-Triazole-3-thione, 5-amino-1,2-dihydro-	38
13.0821	2.4949	Alloaromadendrene	99
15.7529	2.3041	Spiro[4.5]dec-6-en-8-one, 1,7-dimethyl-4-(1-methylethyl)-	38
5.4552	2.2994	Butyrolactone	38
13.7943	1.9582	Naphthalene, 1,2,3,5,6,8a-hexahydro-4,7-dimethyl-1-(1-methylethyl)-, (1S-cis)-	91
13.3966	1.9167	Naphthalene, decahydro-4a-methyl-1-methylene-7-(1-methylethenyl)-, [4aR-(4aα,7α,8aβ)]-	99
8.1913	1.9128	3-(4-Isopropylphenyl)-2-methylpropionaldehyde	45
14.8745	1.6225	2-Butanone, 3-(4-tert-butylphenoxy)-	62
13.4263	1.3525	Longifolene	93
4.2800	1.3414	Benzo[h]quinoline, 2,4-dimethyl-	72
5.6867	1.2760	Pentanoic acid, 3-methyl-	38
9.6455	1.1594	Isobutyramide, N-benzyl-N-phenethyl-	58
14.6965	1.0816	Aromandendrene	95
12.8090	1.0772	Tricyclo[6.3.0.0(2,4)]undec-8-ene, 3,3,7,11-tetramethyl-	95
13.2245	1.0000	2-Isopropenyl-4a,8-dimethyl-1,2,3,4,4a,5,6,7-octahydronaphthalene	99
15.6105	0.8814	Naphthalene, 1,6-dimethyl-4-(1-methylethyl)-	83
13.9842	0.8281	Naphthalene, 1,2,4a,5,6,8a-hexahydro-4,7-dimethyl-1-(1-methylethyl)-, [1S-(1α,4aβ,8aα)]-	95
5.0338	0.8070	2-Butenoic acid, 2-methyl-, (Z)-	38
8.5475	0.7720	2-Amino-6-methylbenzoic acid	30
16.3168	0.7643	7-Acetyl-2-hydroxy-2-methyl-5-isopropylbicyclo[4.3.0]nonane	50
9.6870	0.7307	2H-1,4-Benzoxazin-3(4H)-one	18
15.8954	0.6821	4-Methoxycarbonyl-2-methoxyphenyl isothiocyanate	25
14.0732	0.6636	α-Calacorene	90
4.4462	0.6559	Butanoic acid, 3-methyl-	47
15.0644	0.6500	4a(2H)-Naphthalenecarboxylic acid, 1,3,4,5,6,7-hexahydro-1,1-dimethyl-2-oxo-, ethyl ester	62
5.9894	0.6484	1-[(1H-Pyrrol-2-ylcarbonyl)oxy]-2,5-pyrrolidinedione	35
14.4590	0.6407	1H-1,5-Benzodiazepine, 2,3,4,5-tetrahydro-2,2,4-trimethyl-	52
14.1089	0.6380	5,5'-Di(ethoxycarbonyl)-3,3'-dimethyl-4,4'-dipropyl-2,2'-dipyrrylmethane	38
13.7052	0.5387	Naphthalene, 1,2,4a,5,6,8a-hexahydro-4,7-dimethyl-1-(1-methylethyl)-	95
16.6254	0.5003	2-(Acridin-9-ylamino)-3-phenyl-propionic acid	27
10.1322	0.4990	3H,6H-Thieno[3,4-c]isoxazole, 3a,4-dihydro-6-(1-methylethyl)-	45
4.5411	0.4899	Hexanoic acid, 2-methyl-	32

15.1179	0.4794	8-epi-γ-eudesmol	92
6.9568	0.4633	2-Cyclopenten-1-one, 2-hydroxy-3-methyl-	87
15.0229	0.4247	Megastigmatrienone	95
16.2515	0.4234	N-Methyl-1-adamantaneacetamide	30
11.6279	0.3952	1,2,3-Propatriol, 1-indol-4-yl(ether)	38
16.1091	0.3923	Benzaldehyde, 2-nitro-, diaminomethylidenhydrazone	38
12.1205	0.3793	3-(p-Ethoxyphenyl)-5-(O-tolyloxymethyl)-2-oxazolidone	38
6.7075	0.3664	Cyclohexane-1,3-dione, 2-allylaminomethylene-5,5-dimethyl-	25
6.6363	0.3343	Benzene, 1-methyl-3-(1-methylethyl)-	87
13.2660	0.3266	2-Ethylacridine	18
12.6547	0.3098	Morphinan, 7,8-didehydro-3-methoxy-17-methyl-6-methylene-, (-)-	38
12.5479	0.2966	Naphthalene, 2,6-dimethyl-	86
5.3958	0.2807	1-Methylimidazolidin-2-one	35
10.6723	0.2457	1,2-Benzisothiazol-3-amine tbdms	50
24.1039	0.1529	2-Methyl-7-phenylindole	46

Table S6. Main chemical compounds identified by GC-MS in *C. officinalis* leaf extract under EOD R:FR 2h treatment.

RT (min)	Area (%)	Assignment	Qual
5.0633	45.3190	Oxime-, methoxy-phenyl-_	91
15.3670	8.6110	α-Cadinol	99
13.4915	6.1729	1H-Cycloprop[e]azulene, 1a,2,3,5,6,7,7a,7b-octahydro-1,1,4,7-tetramethyl-, [1aR-(1aα,7α,7aβ,7bα)]-	99
13.3965	4.0126	Naphthalene, decahydro-4a-methyl-1-methylene-7-(1-methylethenyl)-, [4aR-(4aα,7α,8aβ)]-	99
5.8290	3.9835	4-Imidazolidinone, 2-thioxo-	38
15.2187	2.6523	Bicyclo[4.4.0]dec-1-ene, 2-isopropyl-5-methyl-9-methylene-	95
13.0820	2.2386	Alloaromadendrene	99
26.4186	1.9344	Benzo[h]quinoline, 2,4-dimethyl-	52
5.4788	1.6202	5,6-Dimethyl-1,10-phenanthroline	43
19.9788	1.6028	Methyl stearate	99
13.7942	1.5585	Naphthalene, 1,2,3,5,6,8a-hexahydro-4,7-dimethyl-1-(1-methylethyl)-, (1S-cis)-	96
15.7528	1.4433	Isoaromadendrene epoxide	30
14.6964	1.3887	Ledol	95
8.2803	1.3844	Phthalic acid, ethyl hex-3-yl ester	35
12.8089	1.2359	Tricyclo[6.3.0.0(2,4)]undec-8-ene, 3,3,7,11-tetramethyl-	94
18.0676	1.2332	Pentadecanoic acid, 14-methyl-, methyl ester	97
5.6153	1.1928	Heptanoic acid	27
13.6339	1.1387	2-Isopropenyl-4a,8-dimethyl-1,2,3,4,4a,5,6,8a-octahydronaphthalene	94
7.9479	1.1355	13H-Dibenzo[a,i]carbazole	38
13.2244	1.1258	Naphthalene, decahydro-4a-methyl-1-methylene-7-(1-methylethylidene)-, (4aR-trans)-	99
13.9901	1.0270	Naphthalene, 1,2,3,5,6,7,8,8a-octahydro-1,8a-dimethyl-7-(1-methylethenyl)-, [1R-(1α,7β,8aα)]-	93
8.1735	0.9748	1H-1,5-Benzodiazepine, 2,3,4,5-tetrahydro-2,2,4-trimethyl-	50
4.3452	0.9600	Butanoic acid, 3-methyl-	35
14.0672	0.8683	α-Calacorene	94
14.8744	0.8614	3-Buten-2-one, 4-(2,6,6-trimethyl-1-cyclohexen-1-yl)-	70
3.2056	0.7977	2-Propanone, 1-hydroxy-	59
9.6454	0.7038	3-Amino-3-p-tolyl-propionic acid ethyl ester	53
16.3167	0.5350	7-Acetyl-2-hydroxy-2-methyl-5-isopropylbicyclo[4.3.0]nonane	55
4.9328	0.4289	o-(p-Tolyl) 1-azetidinecarbothioate	38
25.6707	0.4113	2-(Acetoxymethyl)-3-(methoxycarbonyl)biphenylene	47
13.7052	0.4052	γ-Muurolene	87
11.5506	0.4034	Thieno[2,3-b]pyridine-2-carboxamide, 3-amino-6-methyl-	37
12.1204	0.3923	Cholestan-7-ol, 8,14-epoxy-3-(phenylmethoxy)-, (3β,5α,7α,8α)-	35
9.5148	0.1789	4-Ethylbenzamide	27
6.6303	0.0680	Benzene, 1-ethyl-2,3-dimethyl-	60

Table S7. Main chemical compounds identified by GC-MS in *C. officinalis* leaf extract under EOD R:FR 4h treatment.

RT (min)	Area (%)	Assignment	Qual
4.9624	37.1298	Oxime-, methoxy-phenyl-_	91
15.3670	23.2741	α-Cadinol	99
13.4914	7.0459	1H-Cycloprop[e]azulene, 1a,2,3,5,6,7,7a,7b-octahydro-1,1,4,7-tetramethyl-, [1aR-(1aα,7α,7aβ,7bα)]-	97
13.3965	5.7770	Naphthalene, decahydro-4a-methyl-1-methylene-7-(1-methylethenyl)-, [4aR-(4aα,7α,8aβ)]-	99
9.6335	3.6398	3-Amino-3-p-tolyl-propionic acid ethyl ester	47
5.8111	2.7976	Pyrido[2,3-d]pyrimidine, 4-phenyl-	38
13.0819	2.6876	Alloaromadendrene	99
4.3451	2.3157	1-(4-Nitrophenyl)piperazine	35
13.7941	2.3064	Naphthalene, 1,2,3,5,6,8a-hexahydro-4,7-dimethyl-1-(1-methylethyl)-, (1S-cis)-	98
13.6339	1.4626	2-Isopropenyl-4a,8-dimethyl-1,2,3,4,4a,5,6,8a-octahydronaphthalene	83
13.9900	1.4542	1,2,4-Metheno-1H-indene, octahydro-1,7a-dimethyl-5-(1-methylethyl)-, [1S-(1α,2α,3aβ,4α,5α,7aβ,8S*)]-	92
14.6963	1.3876	β-Humulene	91
12.8089	1.3159	Tricyclo[6.3.0.0(2,4)]undec-8-ene, 3,3,7,11-tetramethyl-	95
13.2243	1.2664	2-Isopropenyl-4a,8-dimethyl-1,2,3,4,4a,5,6,7-octahydronaphthalene	83
16.3166	1.2505	7-Acetyl-2-hydroxy-2-methyl-5-isopropylbicyclo[4.3.0]nonane	42
26.6856	1.1061	Benzo[h]quinoline, 2,4-dimethyl-	49
4.2383	1.0867	Butanoic acid, 3-methyl-	50
5.7755	0.8571	D-Alanine, N-ethoxycarbonyl-, hexyl ester	27
18.0675	0.7403	Hexadecanoic acid, methyl ester	99
11.5446	0.5581	Phenol, 2,6-dimethoxy-	64
19.9787	0.5408	Methyl stearate	91

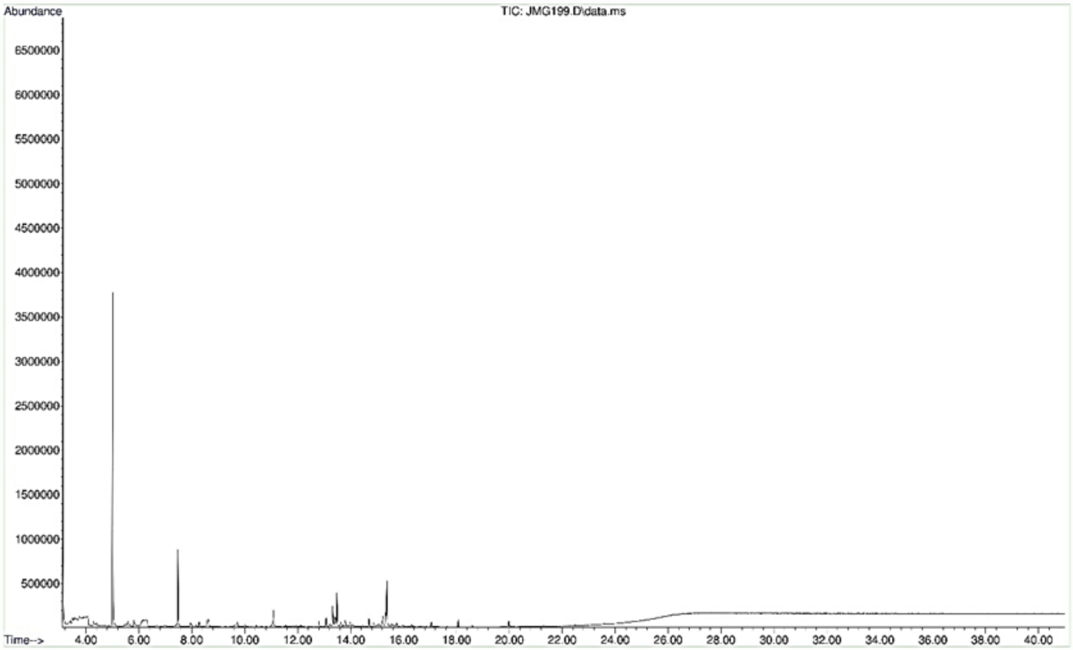

Figure S4. GC–MS chromatogram of *C. officinalis* .leaf hydromethanolic extract under the control treatment.

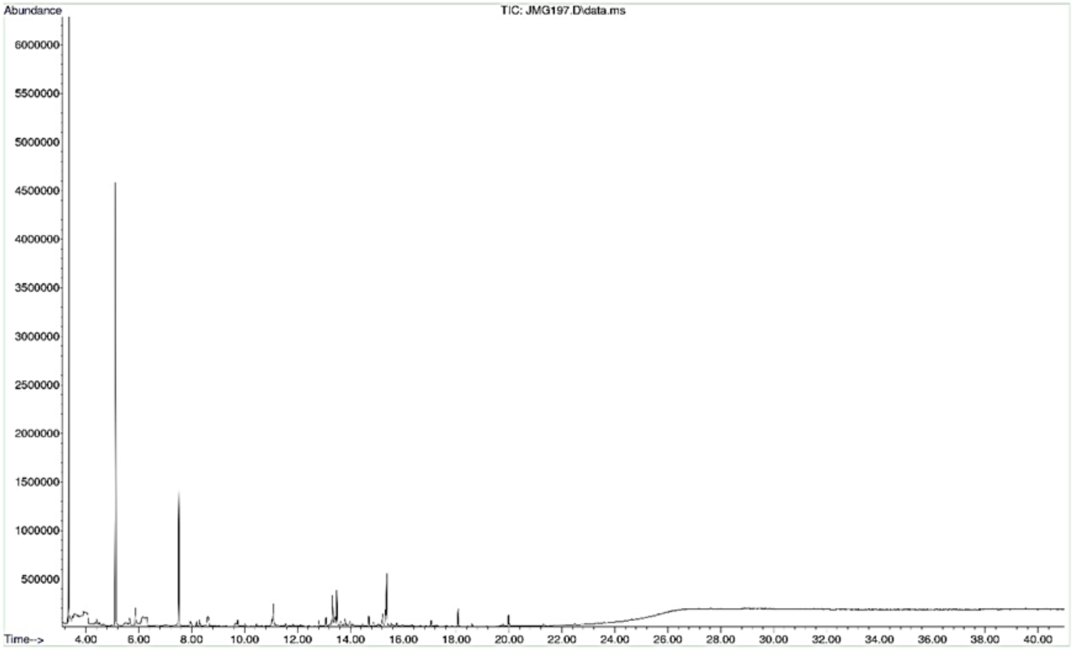

Figure S5. GC–MS chromatogram of *C. officinalis* .leaf hydromethanolic extract under EOD G 2h treatment.

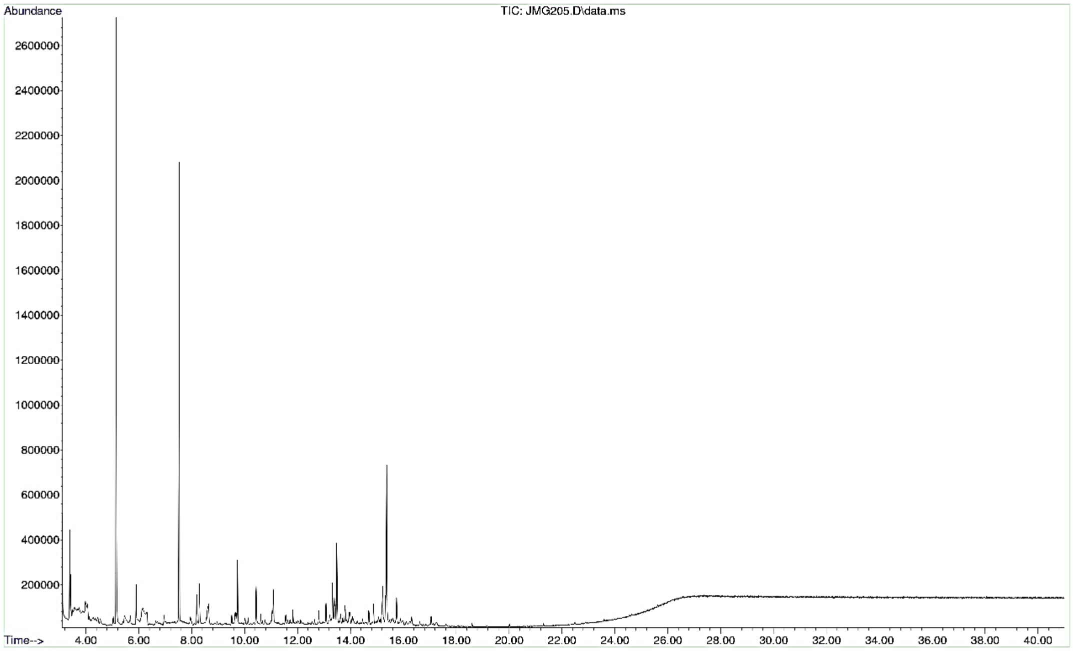

Figure S6. GC–MS chromatogram of *C. officinalis* leaf hydromethanolic extract under EOD G 4h treatment.

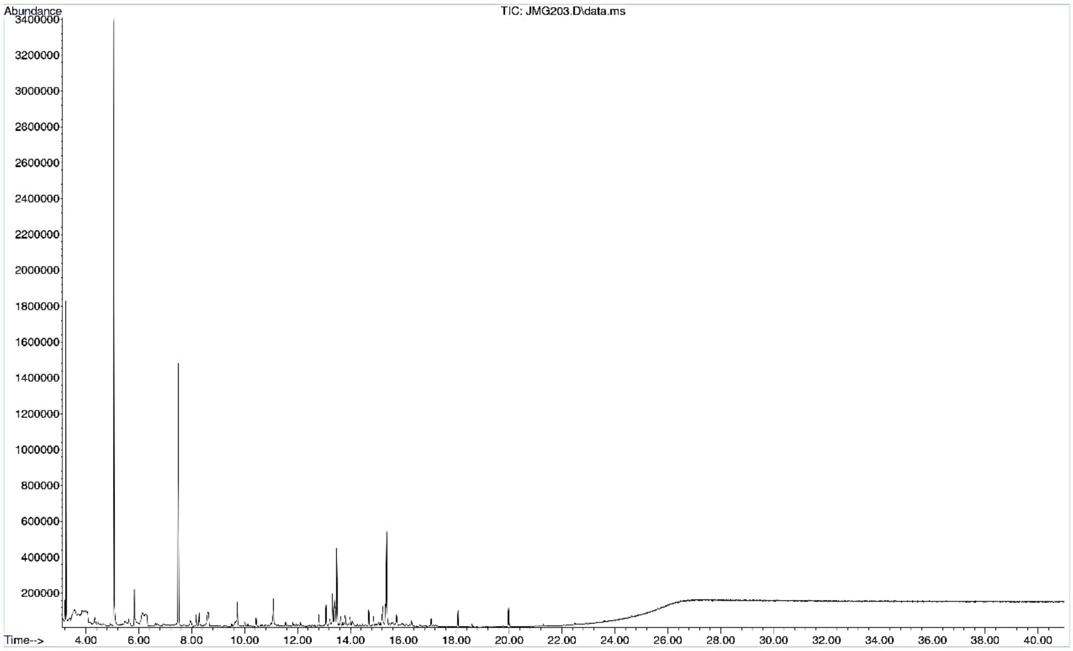

Figure S7. GC–MS chromatogram of *Calendula officinalis L.* leaf hydromethanolic extract under EOD R:FR 2h treatment.

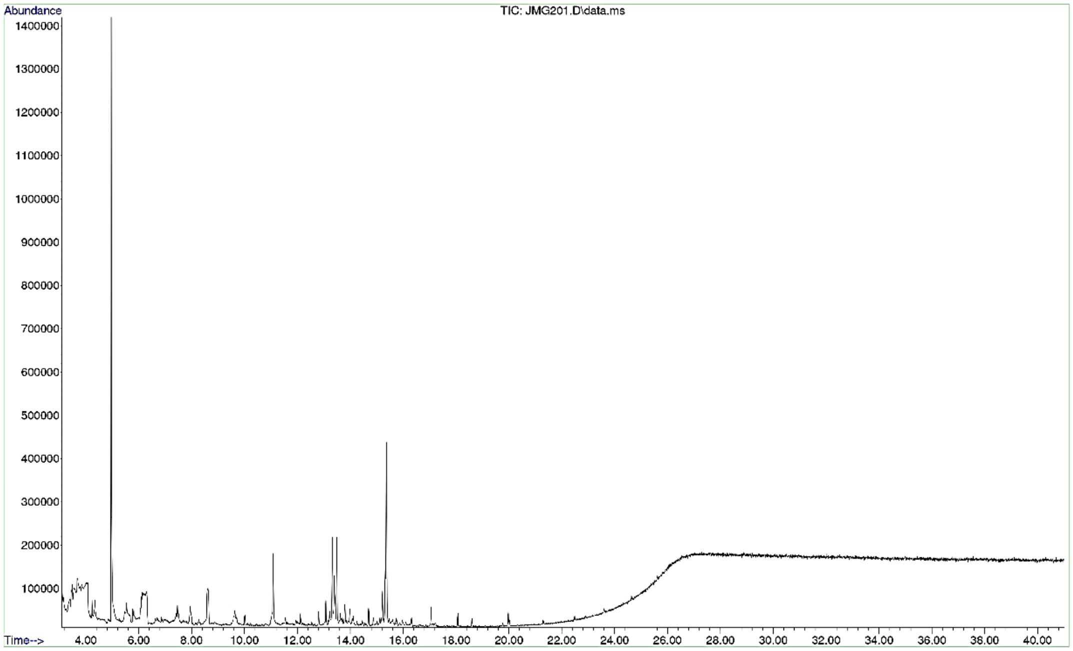

Figure S8. GC–MS chromatogram of *C officinalis* leaf hydromethanolic extract under EOD R:FR 4h treatment.

Table S8. Main chemical compounds identified by GC-MS in *C. officinalis* flower extract under the control treatment.

RT (min)	Area (%)	Assignment	Qual
5.1107	25.8200	Oxime-, methoxy-phenyl-_	91
15.3728	16.6102	1,4-Methano-1H-indene, octahydro-1,7a-dimethyl-4-(1-methylethenyl)-, [1S-(1α,3aβ,4α,7aβ)]-	38
3.3064	7.0881	2-Propanone, 1-hydroxy-	80
3.8821	5.5477	Acetamide	58
13.4914	4.8333	1H-Cycloprop[e]azulene, 1a,2,3,5,6,7,7a,7b-octahydro-1,1,4,7-tetramethyl-, [1aR-(1aα,7α,7aβ,7bα)]-	98
15.2185	4.5629	α-Cadinol	89
5.8704	4.2103	3H-1,2,4-Triazole-3-thione, 5-amino-1,2-dihydro-	38
13.4023	3.8369	Naphthalene, decahydro-4a-methyl-1-methylene-7-(1-methylethenyl)-, [4aR-(4aα,7α,8aβ)]-	99
13.0818	2.2133	Alloaromadendrene	99
13.2243	2.0497	Naphthalene, decahydro-4a-methyl-1-methylene-7-(1-methylethylidene)-, (4aR-trans)-	94
8.1852	1.7039	4-Ethylbenzoic acid, allyl ester	45
13.7941	1.6656	Naphthalene, 1,2,3,5,6,8a-hexahydro-4,7-dimethyl-1-(1-methylethyl)-, (1S-cis)-	96
14.1086	1.5969	Cyclohexanemethanol, 4-ethenyl-α,α,4-trimethyl-3-(1-methylethenyl)-, [1R-(1α,3α,4β)]-	76
13.9899	1.1891	1H-Cycloprop[e]azulene, 1a,2,3,4,4a,5,6,7b-octahydro-1,1,4,7-tetramethyl-, [1aR-(1aα,4α,4aβ,7bα)]-	95
12.8088	1.0067	Tricyclo[6.3.0.0(2,4)]undec-8-ene, 3,3,7,11-tetramethyl-	96
15.5450	0.9613	2,5-Thiophenedicarboxylic acid, tetrahydro-, dimethyl ester, cis-	35
12.1262	0.9237	4-Imino-1,5,5-triphenyl-2-imidazolidinone	27
13.6338	0.9176	2-Isopropenyl-4a,8-dimethyl-1,2,3,4,4a,5,6,8a-octahydronaphthalene	78
4.4044	0.8517	Butanoic acid, 3-methyl-	50
9.6453	0.7804	1-[4-(tert-Butyl)phenyl]-2-(4-toluidino)-1-ethanone	49
16.3165	0.7672	7-Acetyl-2-hydroxy-2-methyl-5-isopropylbicyclo[4.3.0]nonane	60
24.7625	0.7653	Benzo[h]quinoline, 2,4-dimethyl-	49
5.6567	0.7446	Pentanoic acid, 3-methyl-	38
5.4193	0.6915	Butyrolactone	45
13.9424	0.6877	2-(1H-Benzoimidazol-2-yl)-3-phenyl-4H-benzo[1,4]thiazine	35
15.1236	0.6567	2-Naphthalenemethanol, 1,2,3,4,4a,5,6,7-octahydro-α,α,4a,8-tetramethyl-, (2R-cis)-	94
17.1416	0.6228	Adamantane, 1-isothiocyanato-3-methyl-	38
13.7110	0.5411	Naphthalene, 1,2,3,4,4a,5,6,8a-octahydro-7-methyl-4-methylene-1-(1-methylethyl)-, (1α,4aβ,8aα)-	96
6.9328	0.4893	1,2-Cyclopentanedione, 3-methyl-	64
14.8743	0.4847	Acetic acid, 6,6-dimethyl-2-methylene-7-(3-oxobutylidene)oxepan-3-ylmethyl ester	43
11.5446	0.4714	Phenol, 3,4-dimethoxy-	42
10.1260	0.4676	3H,6H-Thieno[3,4-c]isoxazole, 3a,4-dihydro-6-(1-methylethyl)-	72

8.5472	0.4141	4-Methyl-3-(3-nitrophenyl)-6-phenyl-5,6-dihydro-4H-[1,2,4,5]oxatriazine	22
15.4797	0.4095	1H-Indole, 5-methyl-2-phenyl-	30
13.8653	0.4025	1H-3a,7-Methanoazulene, 2,3,4,7,8,8a-hexahydro-3,6,8,8-tetramethyl-, [3R-(3α,3aβ,7β,8aα)]-	78
6.6123	0.3561	Ethanol, 2-(vinyloxy)-	27
4.5053	0.3496	Butanoic acid, 2-methyl-	47
6.4165	0.3152	Erythritol	27
4.9860	0.3044	2(5H)-Thiophenone	46
11.6277	0.3000	1H-Indole-3-carboxylic acid, 5-hydroxy-	22
9.2951	0.2980	Propanal, dimethylhydrazone	30
13.2718	0.2657	3,5-Dimethylbenzaldehyde thiocarbamoylhydrazone	38
18.0675	0.2574	Hexadecanoic acid, methyl ester	91
16.6252	0.2346	1-Propanone, 3-(2-hydroxyphenyl)-1,3-diphenyl-	35
19.9727	0.2213	Methyl stearate	95
21.3022	0.1123	2-Methyl-7-phenylindole	27

Table S9. Main chemical compounds identified by GC-MS in *C. officinalis* flower extract under EOD G 2h treatment.

RT (min)	Area (%)	Assignment	Qual
15.3728	22.3121	2(1H)-Azulenone, 4,5,6,7,8,8a-hexahydro-8a-methyl-, (S)-	53
4.9326	8.5269	Oxime-, methoxy-phenyl-_	91
3.5616	8.5046	Acetamide	58
13.4913	7.7697	1H-Cycloprop[e]azulene, 1a,2,3,5,6,7,7a,7b-octahydro-1,1,4,7-tetramethyl-, [1aR-(1aα,7α,7aβ,7bα)]-	94
15.2244	6.1002	α-Cadinol	91
13.4023	4.8922	Naphthalene, decahydro-4a-methyl-1-methylene-7-(1-methylethenyl)-, [4aR-(4aα,7α,8aβ)]-	99
5.5084	2.9487	Pentanoic acid, 3-methyl-	27
13.7940	2.4794	Naphthalene, 1,2,3,5,6,8a-hexahydro-4,7-dimethyl-1-(1-methylethyl)-, (1S-cis)-	90
16.3165	2.2819	7-Acetyl-2-hydroxy-2-methyl-5-isopropylbicyclo[4.3.0]nonane	46
6.4224	2.2489	Propanamide, 2-hydroxy-	76
7.9537	2.0308	Ether, bis(p-tert-butylphenyl)	43
14.6962	1.8671	Ledol	99
13.0818	1.8559	Alloaromadendrene	99
15.5450	1.7263	2,5-Thiophenedicarboxylic acid, tetrahydro-, dimethyl ester, cis-	43
13.9899	1.5884	Naphthalene, 1,2,3,5,6,7,8,8a-octahydro-1,8a-dimethyl-7-(1-methylethenyl)-, [1R-(1α,7β,8aα)]-	94
6.8379	1.5711	2-Cyclopenten-1-one, 2-hydroxy-3-methyl-	64
13.2243	1.4998	Naphthalene, decahydro-4a-methyl-1-methylene-7-(1-methylethylidene)-, (4aR-trans)-	99
13.6338	1.3881	2-Isopropenyl-4a,8-dimethyl-1,2,3,4,4a,5,6,8a-octahydronaphthalene	94
15.0642	1.3000	4-isopropyl-1,6-dimethyl-1,2,3,4-tetrahydronaphthalene	43
12.8088	1.2476	Selina-3,7(11)-diene	89
14.1086	1.2360	Cyclohexanemethanol, 4-ethenyl-α,α,4-trimethyl-3-(1-methylethenyl)-, [1R-(1α,3α,4β)]-	81
9.6215	1.0717	Benzofuran, 2,3-dihydro-	53
6.9981	1.0625	2-Ethylacridine	38
15.7527	1.0523	1,4-Methanoazulen-7(1H)-one, octahydro-4,8,8,9-tetramethyl-, (+)-	43
11.0401	0.9334	2-Methoxy-4-vinylphenol	42
12.1262	0.8576	1-(3-n-Propoxyphenyl)-2-propanone oxime	35
4.1848	0.8441	Butanoic acid, 3-methyl-	76
14.0730	0.8420	α-Calacorene	38
5.2294	0.7903	2-Cyano-3,5-dimethylpyrazine	43
17.1415	0.7355	2-Naphthalenemethanol, decahydro-α,α,4a-trimethyl-8-methylene-, [2R-(2α,4aα,8aβ)]-	90
13.7050	0.7224	Naphthalene, 1,2,3,4,4a,5,6,8a-octahydro-7-methyl-4-methylene-1-(1-methylethyl)-, (1α,4aβ,8aα)-	96
15.1236	0.6619	8-epi-γ-eudesmol	90
14.8743	0.6208	3-Buten-2-one, 4-(2,6,6-trimethyl-1-cyclohexen-1-yl)-	64
14.9455	0.6082	Indane, 2-methoxy-3-(2-methyl-1-propenyl-1)-	38
11.5386	0.5633	Phenol, 2,6-dimethoxy-	89

13.8653	0.5561	Epizonarene	80
22.4833	0.5455	1H-Indole-2-carboxylic acid, 6-(4-ethoxyphenyl)-3-methyl-4-oxo-4,5,6,7-tetrahydro-, isopropyl ester	43
16.6252	0.5388	1H-Indole, 5-methyl-2-phenyl-	35
15.6281	0.5330	Indole-2-one, 2,3-dihydro-N-hydroxy-4-methoxy-3,3-dimethyl-	35
8.1555	0.3367	Cyclopentanone, dimethylhydrazone	38
4.2976	0.2612	Butanoic acid, 2-methyl-	37
12.6545	0.2456	Pyrido[2,3-d]pyrimidine, 4-phenyl-	38
26.7152	0.2413	1,2,4-Benzenetricarboxylic acid, 4-dodecyl dimethyl ester	47

Table S10. Main chemical compounds identified by GC-MS in *C. officinalis* flower extract under EOD G 4h treatment.

RT (min)	Area (%)	Assignment	Qual
5.0930	29.5026	Oxime-, methoxy-phenyl-_	91
15.3671	18.9882	α-Cadinol	99
13.4915	5.8436	1H-Cycloprop[e]azulene, 1a,2,3,5,6,7,7a,7b-octahydro-1,1,4,7-tetramethyl-, [1aR-(1aα,7α,7aβ,7bα)]-	97
3.2828	5.6715	2-Propanone, 1-hydroxy-	80
5.8587	5.4805	4-Imidazolidinone, 2-thioxo-	38
13.4025	4.0676	Naphthalene, decahydro-4a-methyl-1-methylene-7-(1-methylethenyl)-, [4aR-(4aα,7α,8aβ)]-	99
8.2803	2.1058	4-Propylbenzaldehyde diethyl acetal	38
14.1088	1.9659	3,7-Cyclodecadiene-1-methanol, α,α,4,8-tetramethyl-, [s-(Z,Z)]	90
6.2683	1.9597	7H-Dibenzo[b,g]carbazole, 7-methyl-	59
8.1794	1.7868	3-(4-Isopropylphenyl)-2-methylpropionaldehyde	42
13.7942	1.7631	Naphthalene, 1,2,3,5,6,8a-hexahydro-4,7-dimethyl-1-(1-methylethyl)-, (1S-cis)-	98
13.0820	1.7258	Aromandendrene	99
12.1264	1.3967	Cyclohexane, 1-ethenyl-1-methyl-2,4-bis(1-methylethenyl)-, [1S-(1α,2β,4β)]-	98
4.3630	1.3072	Butanoic acid, 3-methyl-	50
15.5451	1.1300	2,5-Thiophenedicarboxylic acid, tetrahydro-, dimethyl ester, cis-	38
13.2244	1.1011	Naphthalene, decahydro-4a-methyl-1-methylene-7-(1-methylethylidene)-, (4aR-trans)-	98
13.9901	1.0894	Naphthalene, 1,2,3,5,6,7,8,8a-octahydro-1,8a-dimethyl-7-(1-methylethenyl)-, [1R-(1α,7β,8aα)]-	94
12.8089	1.0869	Naphthalene, 1,2,3,5,6,7,8,8a-octahydro-1,8a-dimethyl-7-(1-methylethenyl)-, [1S-(1α,7α,8aα)]-	91
25.6233	0.9950	2-(Acetoxymethyl)-3-(methoxycarbonyl)biphenylene	53
13.6340	0.9621	2-Isopropenyl-4a,8-dimethyl-1,2,3,4,4a,5,6,8a-octahydronaphthalene	93
5.6391	0.8811	Pentanoic acid, 3-methyl-	22
14.6964	0.8570	Ledol	99
16.3167	0.6893	7-Acetyl-2-hydroxy-2-methyl-5-isopropylbicyclo[4.3.0]nonane	91
13.7052	0.6570	Naphthalene, 1,2,3,4,4a,5,6,8a-octahydro-7-methyl-4-methylene-1-(1-methylethyl)-, (1α,4aβ,8aα)-	96
13.9426	0.6203	2-(1H-Benzoimidazol-2-yl)-3-phenyl-4H-benzo[1,4]thiazine	35
15.7469	0.5805	3,5-Ethanoquinolin-10-one, decahydro-1,7-dimethyl-, [3R-(3α,4aβ,5α,7β,8aβ)]-	25
11.5507	0.5089	α-Cubebene	60
5.4076	0.5075	Acetaldehyde, dimethylhydrazone	27
18.0676	0.5008	Hexadecanoic acid, methyl ester	98
4.4639	0.4984	Butanoic acid, 2-methyl-	43
15.1237	0.4405	γ-eudesmol	97
15.8894	0.4162	Thiocyanic acid carbazol-3,6-diyl ester	35
19.9788	0.3798	Methyl stearate	98
10.0193	0.3733	4H-3,1-Benzoxazine, 6,7-dimethoxy-2-(4-methoxyphenyl)-4-propyl-	35

14.8744	0.3514	Acetic acid, 2,6,6-trimethyl-3-methylene-7-(3-oxobutylidene)oxepan-2-yl ester	46
6.6362	0.3353	Benzene, 2-ethyl-1,3-dimethyl-	15
4.9684	0.3238	2-Butenoic acid, 2-methyl-, (Z)-	49
13.8654	0.3175	Ylangene	52
11.6278	0.2950	Pyrido[3,2-d]pyrimidin-4(3H)-one, 3-hydroxy-2-methyl-	38
13.2660	0.2170	2-(3-Isopropyl-4-methyl-pent-3-en-1-ynyl)-2-methyl-cyclobutanone	50
5.3542	0.2160	Tiglic acid	35
6.9330	0.1039	1,2-Cyclopentanedione, 3-methyl-	50

Table S11. Main chemical compounds identified by GC-MS in *C. officinalis* flower extract under EOD R:FR 2h treatment.

RT (min)	Area (%)	Assignment	Qual
15.3728	16.8364	2(1H)-Azulenone, 4,5,6,7,8,8a-hexahydro-8a-methyl-, (S)-	56
3.1579	10.6782	2-Propanone, 1-hydroxy-	80
5.0335	8.6517	Oxime-, methoxy-phenyl-_	91
13.4913	5.5269	1H-Cycloprop[e]azulene, 1a,2,3,5,6,7,7a,7b-octahydro-1,1,4,7-tetramethyl-, [1aR-(1aα,7α,7aβ,7bα)]-	97
15.2244	4.6218	Bicyclo[4.4.0]dec-1-ene, 2-isopropyl-5-methyl-9-methylene-	93
13.4023	3.9522	Naphthalene, decahydro-4a-methyl-1-methylene-7-(1-methylethenyl)-, [4aR-(4aα,7α,8aβ)]-	99
3.7633	3.6794	Acetamide	58
8.2682	2.9566	Phthalic acid, ethyl hex-3-yl ester	35
13.0818	2.5621	Alloaromadendrene	99
13.2242	1.6387	Naphthalene, decahydro-4a-methyl-1-methylene-7-(1-methylethylidene)-, (4aR-trans)-	99
13.7940	1.6295	Naphthalene, 1,2,3,5,6,8a-hexahydro-4,7-dimethyl-1-(1-methylethyl)-, (1S-cis)-	94
4.3153	1.4938	Butanoic acid, 3-methyl-	58
15.0642	1.4702	β-Nootkatol	35
6.9981	1.4435	Benzeneacetaldehyde	55
6.8913	1.3683	1,2-Cyclopentanedione, 3-methyl-	94
13.9899	1.3029	1H-Cycloprop[e]azulene, 1a,2,3,4,4a,5,6,7b-octahydro-1,1,4,7-tetramethyl-, [1aR-(1aα,4α,4aβ,7bα)]-	98
3.3301	1.2725	2-Butanol	43
6.4758	1.1846	Propanamide, 2-hydroxy-	50
7.3067	1.1366	2-Ethylacridine	27
7.9418	1.1163	5H-Naphtho[2,3-b]carbazole	46
7.7697	1.1015	1,1-Dimethyl-4-methylenecyclohexane	42
12.8088	1.1012	Aromandendrene	87
15.5449	1.0932	2,5-Thiophenedicarboxylic acid, tetrahydro-, dimethyl ester, cis-	43
14.6962	0.9504	β-Humulene	95
16.3165	0.9476	Benzenamine, 2,5-dihydromethyl-	64
14.1086	0.9423	Cyclohexanemethanol, 4-ethenyl-α,α,4-trimethyl-3-(1-methylethenyl)-, [1R-(1α,3α,4β)]-	62
9.2772	0.8876	(-)-cis-3,4-Dimethyl-2-phenyltetrahydro-1,4-thiazine	35
15.7467	0.8493	9-Isopropyl-1-methyl-2-methylene-5-oxatricyclo[5.4.0.0(3,8)]undecane	90
6.5411	0.8477	α-Piperidinomethyl-6-benzothiazole methanol	38
5.5855	0.8460	Pentanoic acid, 3-methyl-	35
17.1415	0.8055	1H-Indene, 1-ethylideneoctahydro-7a-methyl-, (1E,3aα,7aβ)-	42
6.3452	0.7749	1,2,3,4-Butanetetrol, [S-(R*,R*)]-	35
5.3362	0.7557	Butyrolactone	83
15.1235	0.7372	γ-eudesmol	98
5.4727	0.6777	5,6-Dimethyl-1,10-phenanthroline	47
12.1203	0.6687	Cyclohexanecarboxamide, N-furfuryl-	38
4.9148	0.6616	Tiglic acid	64

14.0730	0.6577	α-Calacorene	94
14.8742	0.6426	4(1H)-Pteridinone, 2-amino-6-methyl-	47
5.9535	0.6370	Phenol	38
5.3896	0.6291	1H-Indole-3-carboxylic acid, 5-hydroxy-	38
13.7109	0.5894	γ-Muurolene	95
4.4162	0.5806	9-Phosphabicyclo[4.2.1]nona-2,4,7-trien-9-amine, N,N-diethyl-, syn-	50
11.5386	0.5573	Phenol, 2,6-dimethoxy-	70
14.5656	0.4931	2-Methyl-4-(2,6,6-trimethylcyclohex-1-enyl)but-2-en-1-ol	20
15.6221	0.4465	3,5-Dimethylbenzaldehyde thiocarbamoylhydrazone	18
18.2218	0.4464	Benzaldehyde, 2-nitro-, diaminomethylidenhydrazone	46
12.9631	0.4439	1,4-Phthalazinedione, 2,3-dihydro-6-nitro-	38
14.4647	0.4419	Diisopropylketone p-tosylhydrazone	25
16.2334	0.4274	Ethanone, 1-[3-[[5-(3,3-dimethyloxiranyl)-3-methyl-2-pentenyl]oxy]phenyl]-, (E)-.+/-.-	17
16.6192	0.4197	2-Benzothiazolamine, 5,6-dimethyl-	44
17.2187	0.4114	6-Isopropenyl-4,8a-dimethyl-1,2,3,5,6,7,8,8a-octahydro-naphthalen-2-ol	35
9.6334	0.3900	Benzofuran, 2,3-dihydro-	46
13.8652	0.3627	Epizonarene	92
11.6276	0.3598	Pyrido[3,2-d]pyrimidin-4(3H)-one, 3-hydroxy-2-methyl-	38
17.6045	0.3566	9-Amino-7-mercapto-5,6,8,10-tetraaza-benzo[b]fluoren-11-one	35
10.0132	0.3231	4H-3,1-Benzoxazine, 6,7-dimethoxy-2-(4-methoxyphenyl)-4-propyl-	38
9.4969	0.2542	Adamantane, 1-isothiocyanato-3-methyl-	14
10.1201	0.2515	3H,6H-Thieno[3,4-c]isoxazole, 3a,4-dihydro-6-(1-methylethyl)-	50
5.2828	0.2274	1-Methylimidazolidin-2-one	38
18.0674	0.2096	Hexadecanoic acid, methyl ester	96
19.7768	0.1487	5-Acetamido-4,7-dioxo-4,7-dihydrobenzofurazan	22
8.9389	0.1221	Benzoic acid, 2-(methylamino)-, 2-methylpropyl ester	46

Table S12. Main chemical compounds identified by GC-MS in *C. officinalis* flower extract under EOD R:FR 4h treatment.

RT (min)	Area (%)	Assignment	Qual
15.3671	21.0449	α-Cadinol	99
5.0752	18.9604	Oxime-, methoxy-phenyl-_	80
13.4915	7.0014	1H-Cycloprop[e]azulene, 1a,2,3,5,6,7,7a,7b-octahydro-1,1,4,7-tetramethyl-, [1aR-(1aα,7α,7aβ,7bα)]-	94
3.2294	6.4120	2-Propanone, 1-hydroxy-	80
13.4025	4.1754	Naphthalene, decahydro-4a-methyl-1-methylene-7-(1-methylethenyl)-, [4aR-(4aα,7α,8aβ)]-	99
3.8348	2.9843	Acetamide	49
8.2803	2.5914	Phthalic acid, cyclobutyl ethyl ester	35
13.0820	1.9033	Alloaromadendrene	99
13.7942	1.7753	Naphthalene, 1,2,3,5,6,8a-hexahydro-4,7-dimethyl-1-(1-methylethyl)-, (1S-cis)-	98
5.8468	1.6464	4-Imidazolidinone, 2-thioxo-	35
4.3571	1.3002	Butanoic acid, 3-methyl-	50
18.0676	1.2922	Hexadecanoic acid, methyl ester	99
13.9901	1.2510	1H-Cycloprop[e]azulene, 1a,2,3,4,4a,5,6,7b-octahydro-1,1,4,7-tetramethyl-, [1aR-(1aα,4α,4aβ,7bα)]-	98
19.9729	1.2398	Methyl stearate	98
16.3167	1.1565	7-Acetyl-2-hydroxy-2-methyl-5-isopropylbicyclo[4.3.0]nonane	81
8.1794	1.1525	1H-1,5-Benzodiazepine, 2,3,4,5-tetrahydro-2,2,4-trimethyl-	50
15.5451	1.1436	4-Methoxy-3-buten-2-one	35
15.0644	1.1084	Ethanone, 1-(1,3a,4,5,6,7-hexahydro-4-hydroxy-3,8-dimethyl-5-azulenyl)-	25
13.2244	1.1038	Naphthalene, decahydro-4a-methyl-1-methylene-7-(1-methylethylidene)-, (4aR-trans)-	99
12.8089	1.0896	Tricyclo[6.3.0.0(2,4)]undec-8-ene, 3,3,7,11-tetramethyl-	95
26.5670	1.0735	1,4-Benzenediol, 2,5-bis(1,1-dimethylethyl)-	47
13.6340	1.0645	Naphthalene, 1,2,3,5,6,7,8,8a-octahydro-1,8a-dimethyl-7-(1-methylethenyl)-, [1S-(1α,7α,8aα)]-	86
4.9566	0.9300	Tiglic acid	78
5.3780	0.8156	Butyrolactone	64
5.4848	0.8081	5,6-Dimethyl-1,10-phenanthroline	47
15.8894	0.7856	Acetamide, 2-[4-(1-oxo-3-phenyl-2-propenyl)phenyloxy]-	50
5.6213	0.7541	Pentanoic acid, 3-methyl-	35
14.1088	0.7476	Cyclohexanemethanol, 4-ethenyl-α,α,4-trimethyl-3-(1-methylethenyl)-, [1R-(1α,3α,4β)]-	49
15.7529	0.7196	9-Isopropyl-1-methyl-2-methylene-5-oxatricyclo[5.4.0.0(3,8)]undecane	74
6.4938	0.6274	Propanamide, 2-hydroxy-	50
14.8745	0.6160	Benzenepropanamide, N,N-dimethyl-	38
15.9725	0.6143	Tridecanoic acid, 12-methyl-, methyl ester	91
14.0732	0.5559	Cadala-1(10),3,8-triene	46
15.1237	0.5536	γ-eudesmol	97
6.9093	0.5324	1,2-Cyclopentanedione, 3-methyl-	93

12.1205	0.5304	2-Methyl-6-(5-methyl-2-thiazolin-2-ylamino)pyridine	25
15.6282	0.5176	Purin-2,6-dione, 1,3-dimethyl-8-[2-nitrophenethenyl]-	25
17.1417	0.5133	2-Naphthalenemethanol, decahydro-α,α,4a-trimethyl-8-methylene-, [2R-(2α,4aα,8aβ)]-	46
13.7052	0.5083	Naphthalene, 1,2,4a,5,6,8a-hexahydro-4,7-dimethyl-1-(1-methylethyl)-	96
14.9516	0.4874	Cycloisolongifolene, 8,9-dehydro-	38
7.0102	0.4797	Benzeneacetaldehyde	55
19.7770	0.4794	9,12,15-Octadecatrienoic acid, methyl ester, (Z,Z,Z)-	95
15.4799	0.4653	N-Methyl-1-adamantaneacetamide	20
14.5599	0.4360	Hexanoic acid, 2,7-dimethyloct-7-en-5-yn-4-yl ester	27
11.5447	0.4343	Phenol, 2,6-dimethoxy-	42
4.4580	0.4332	Butanoic acid, 2-methyl-	47
13.8654	0.4113	Naphthalene, 1,2,3,4,4a,5,6,8a-octahydro-7-methyl-4-methylene-1-(1-methylethyl)-, (1α,4aβ,8aα)-	94
5.9240	0.3771	Cyclohexane-1,3-dione, 2-allylaminomethylene-5,5-dimethyl-	25
16.6254	0.3580	Benzaldehyde, 2-nitro-, diaminomethylidenhydrazone	43
10.0134	0.3423	3-(6-Methyl-3-pyridyl)-1,5-di(p-tolyl)-2-pyrazoline	35
12.9633	0.3181	2-Ethylacridine	46
5.9893	0.2976	Ethyl n-butyl disulphide	22
9.2834	0.2971	Piperazine, 2-methyl-	25
17.2189	0.2923	Pyrido[2,3-d]pyrimidine, 4-phenyl-	14
11.6278	0.2499	Pyrido[3,2-d]pyrimidin-4(3H)-one, 3-hydroxy-2-methyl-	25
19.7117	0.2409	Methyl 10-trans,12-cis-octadecadienoate	91

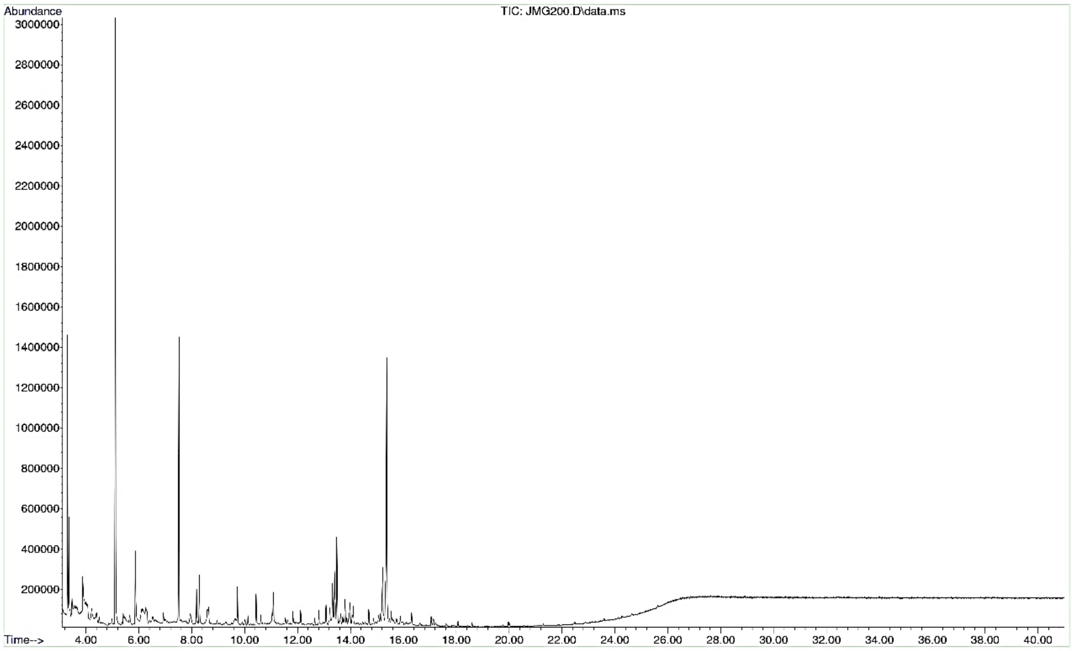

Figure S9. GC–MS chromatogram of *C. officinalis* flower hydromethanolic extract under the control treatment.

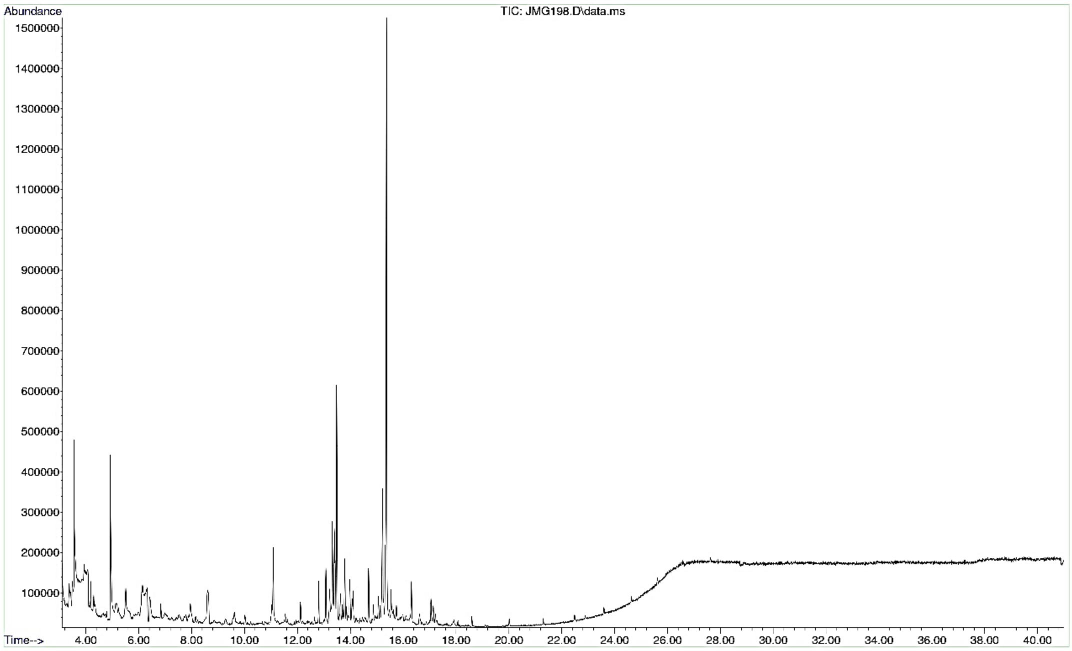

Figure S10. GC–MS chromatogram of *C. officinalis* flower hydromethanolic extract under EOD G 2h treatment.

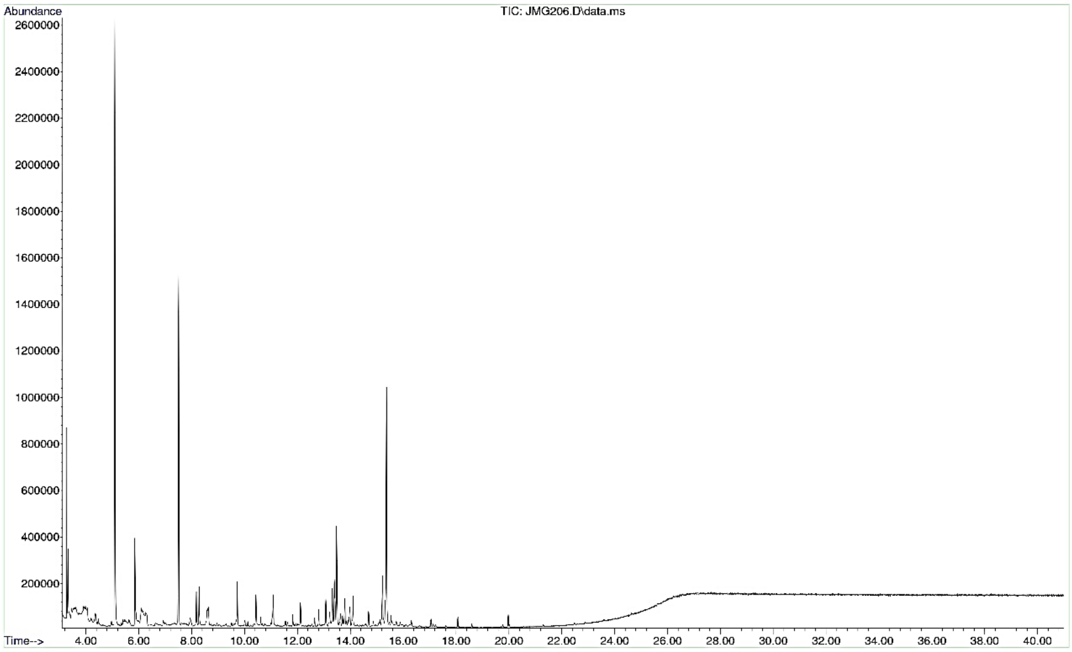

Figure S11. GC–MS chromatogram of *C. officinalis* flower hydromethanolic extract under EOD G 4h treatment.

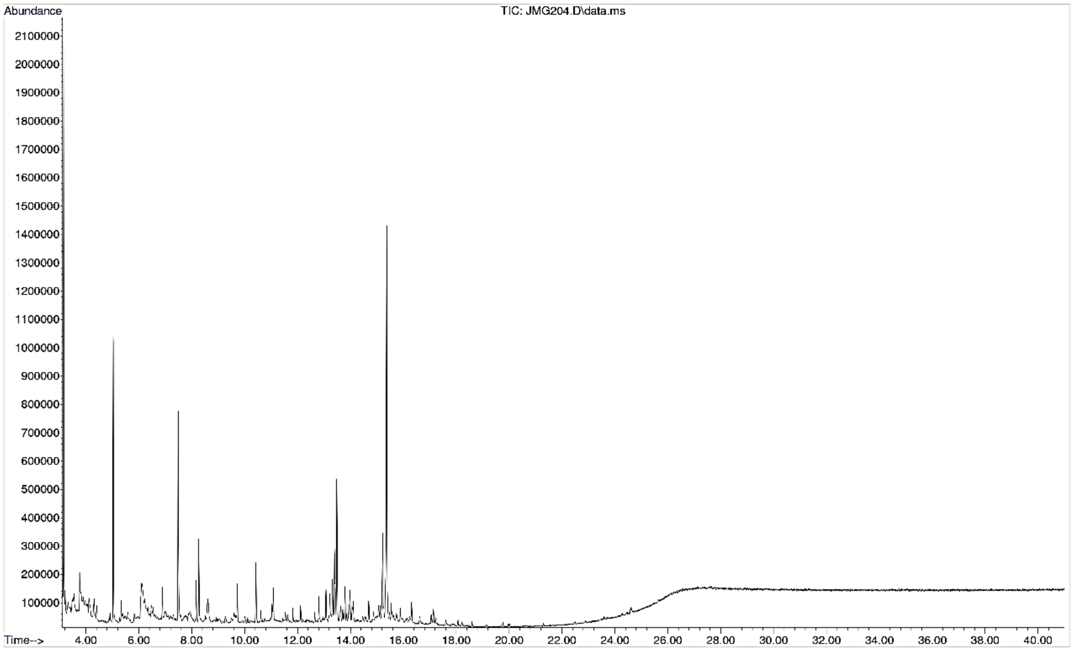

Figure S12. GC–MS chromatogram of *C. officinalis* flower hydromethanolic extract under EOD R:FR 2h treatment.

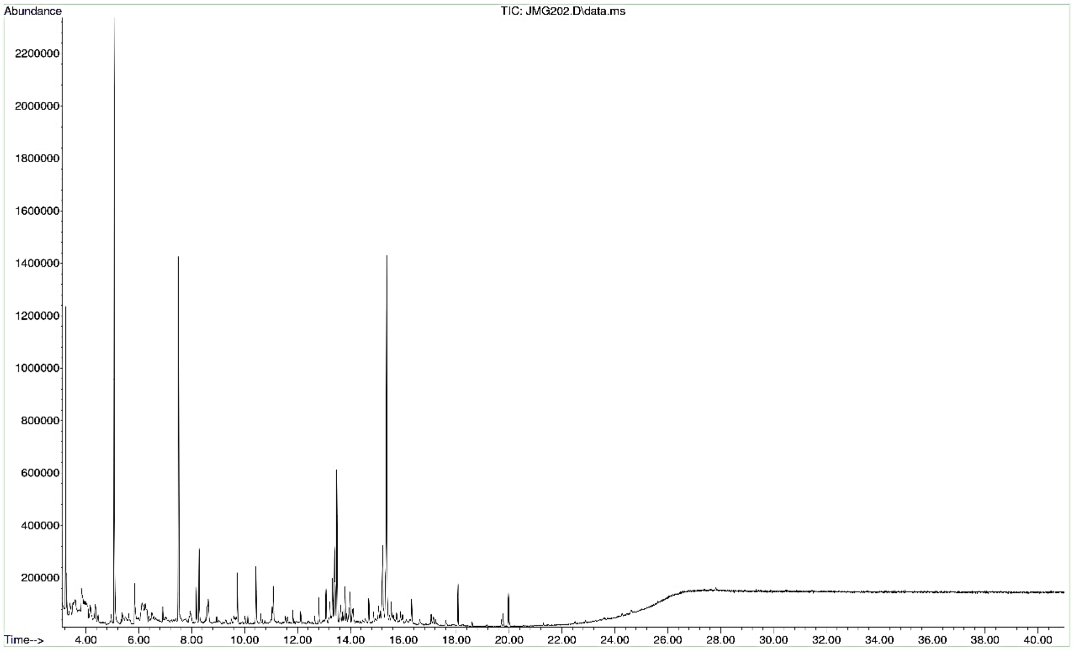

Figure S13. GC–MS chromatogram of *C. officinalis* flower hydromethanolic extract under EOD R:FR 4h treatment.
